# Laser Ablation APCI-HRMS Method for the Analysis of Cultural Heritage Materials

**DOI:** 10.1021/jasms.5c00308

**Published:** 2025-12-18

**Authors:** Anu Teearu, Martin Leissoo, Rynno Lohmus, Alexey Treshchalov, Tõiv Haljasorg, Victor Augusto Xavier da Silveira, Hilkka Hiiop, Signe Vahur

**Affiliations:** † Institute of Chemistry, 37546 University of Tartu, Ravila 14A, 50411 Tartu, Estonia; ‡ Institute of Physics, University of Tartu, W. Ostwaldi 1, 50411 Tartu, Estonia; § 354526 Agilent Technologies Deutschland GmbH, Hewlett-Packard-Str. 8, D-76337 Waldbronn, Germany; ∥ Department of Cultural Heritage and Conservation, Estonian Academy of Arts, Põhja pst 7, 10412 Tallinn, Estonia

**Keywords:** laser ablation, mass spectrometry, high-resolution, cultural heritage

## Abstract

Analyzing cultural heritage (CH) materials, particularly organic substances, is challenging due to their complex chemical composition. A key requirement in such analyses is the use of analytical techniques that cause minimal damage to the artifact while providing the maximum amount of chemical information about the materials. Chromatographic and mass spectrometric (MS) techniques give valuable information about components of the organic materials, but these typically require a microsample from the object, along with specific preparation and instrumental conditions. For CH artifacts, techniques that work directly on the surface, thereby causing minimal damage, are far more desirable. We have developed a 355 nm optical fiber-coupled laser ablation (LA) atmospheric pressure chemical ionization (APCI)-MS system that enables the analysis of organic material directly from the solid surface of the artifact under ambient conditions with minimal surface damage. In this study, we coupled LA with APCI-Fourier transform ion cyclotron resonance (FT-ICR)-MS. The main aim was to evaluate the effectiveness of the developed LA-APCI high-resolution (HR)­MS system for the analysis of five handmade mock-up materials of different paint and varnish layers and one real-life sample. The results demonstrate the analytical potential of the LA-APCI-HRMS technique, as high-quality and identifiable mass spectra were obtained for most of the analyzed materials. In the future, the developed LA-APCI-HRMS technique could be applied not only to cultural heritage but also to other fields (e.g., forensics, material science, etc.).

## Graphical abstract

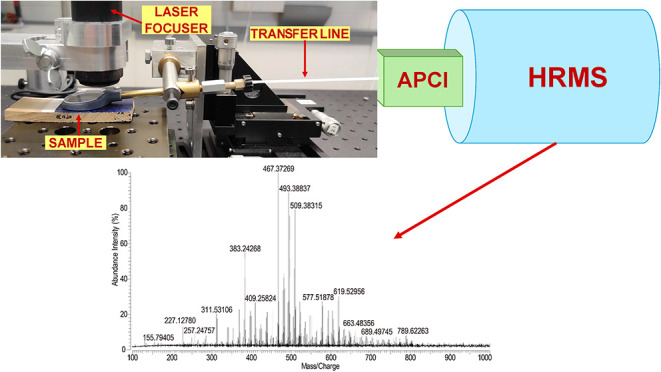

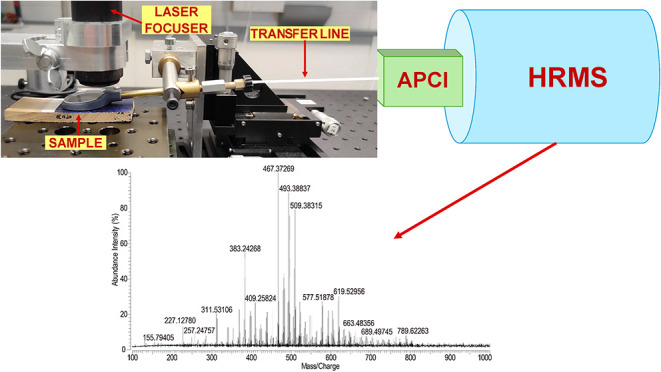

## 1 Introduction

Chemical analysis of cultural heritage (CH) objects (like paintings, sculptures, manuscripts, textiles, archeological items, etc.) is essential for art historians and archeologists, helping to get information about the authorship, origin, authenticity, and age of the artifacts but also trade of the materials in different historical periods and for conservators to form decisions about the conservation and preservation of objects.

Materials of CH objects, like paints, varnishes, textile dyes, etc., are complex multicomponent and multilayered mixtures. They have undergone aging, and their composition, due to degradation, oxidation, and polymerization, has further changed and becomes even more complicated.^1^ All this makes chemical analysis of such organic materials and their compounds challenging. For the analysis of CH materials, non- or minimally destructive methods are preferred, enabling a quick and direct analysis on the solid surface of the artifact without removal of the sample piece and any sample preparation. This, however, implies limitations for the selection of suitable analytical methods that can be used for the analysis of components of organic materials.

Mass spectrometry (MS) occupies a prominent place among the techniques used for the analysis of CH organic materials. MS offers a wealth of detailed qualitative and quantitative information about compounds of different materials and can do so with low detection limits. The most widely used MS techniques, such as gas chromatography (GC)-MS (also pyrolyser (py)-GC-MS), liquid chromatography (LC)-MS, and direct MS techniques (such as electrospray ionization (ESI)-, atmospheric pressure chemical ionization (APCI)-, matrix-assisted laser desorption/ionization (MALDI)-, direct temperature-resolved (DT)-MS, etc.) are destructive, typically require small sample piece and sometimes a specific sample preparation (e.g., solvent extractions, derivatization, and/or matrix material addition for the ionization) and certain instrumental conditions (e.g., vacuum, gases, temperature, conductivity, etc.).^1–5^

Based on this, ambient ionization mass spectrometry (AIMS) techniques are promising methods, involving direct sampling and ionizing compounds under ambient conditions, allowing rapid, real-time, high-throughput, as well as in situ solids analysis with minimal or no sample preparation.^6,7^ In the analysis of organic CH materials, direct analysis in real time (DART)-MS and desorption electrospray ionization (DESI)-MS have seen growing adoption.^3,8–12^ These methods, however, also have drawbacks. DART-MS is a plasma-based technique that uses a quite large spot size (about 1 mm in diameter) and elevated gas temperature (about several hundred degrees), which may cause thermal desorption and fragmentation of the surface of some of the materials.^8,13,14^ Also, in a typical setup, the sample must be positioned between the DART source and the MS inlet (distance approximately 1 cm); therefore, it is more suitable for the smaller objects or samples, and analysis is problematic with larger objects.^8,15^ However, DART-MS has been modified and a 30 cm long transfer tube has been exploited to perform surface analysis from somewhat larger objects.^9,16^ DESI-MS is a liquid extraction-based AIMS method that applies a continuous spray of solvent to dissolve material components (suitable only for specific CH materials) from the object’s surface, and the used solvents may somewhat damage the object’s surface.^8,10,17^ For the analysis of larger CH objects, an in-house AIMS technique called nonproximate desorption photoionization (NPDPI) MS has been developed.^15^ In this technique, the molecules are desorbed from the object’s surface with a heated jet of inert N_2_ gas and ionized in the heated transport tube (approximately 2 m long) by a vacuum UV lamp (photoionization). It is a soft ionization technique that leaves practically no marks on the analyzed surface. However, this robust and complex system is in the development phase and is mostly dedicated to the analysis of objects with smoother surfaces and more volatile compounds.

There are also laser-based AIMS techniques available, like matrix-assisted laser desorption electrospray ionization (MALDESI)-MS, laser ablation electrospray ionization (LAESI)-MS, electrospray laser desorption ionization (ELDI)-MS, and laser ablation atmospheric pressure chemical ionization (LA-APCI)-MS, which have been tested for the analysis of organic materials.^18^ All these laser-based techniques are not entirely suitable for analyzing CH organic materials. For example, in MALDESI-MS, the analyzed material must be mixed with matrix material (or contain water in case an IR laser is used) and placed on a conductive plate near the MS inlet.^18,19^ In LA-APCI-MS, the sample must fit into a specific sample chamber purged with N_2_ gas.^20^ LAESI-MS uses an IR laser, which is not suitable for ablating most of the CH materials (only applicable for water-containing substances).^18,19^ In ELDI-MS, the sample must be close to the MS inlet or in a specific ablation cell.^19,21^ There are laser-based AIMS instruments with transfer lines for transporting the ablated sample components, but these systems are mostly developed for very specific applications (e.g., tissue studies, metabolomics) and are not easily adaptable and suitable for the analysis of CH materials.^21–23^ In most of the developed laser-based AIMS instruments, the laser is directed onto the analyzed surface via a complex and bulky optical system, again significantly hindering their application for the analysis of large and uneven-surfaced CH objects.^18–23^ Only a few fiber-coupled laser-based AIMS techniques have been developed. For example, fiber-coupled LAESI-MS (f-LAESI-MS), which allows the analysis of very small areas on the object’s surface (spot size in μm range), but this is achieved only after complex fiber tip preparation (mechanical and solvent treatment, etching with acid).^24,25^ Additionally, the analyzed object must be close to the MS inlet to avoid loss of ions.

Therefore, none of the MS techniques described above are fully suitable for analyzing the organic materials on the solid surfaces of CH objects without removing a sample piece or performing specific sample preparation or requiring specific conditions for the analysis.

We have developed a laser ablation (LA)-APCI-MS system operating in the UV region (355 nm), in which the laser output is delivered to the sample by a flexible 2 m optical fiber ending with a focuser and ablation products can be transported under reduced pressure (without additional gas purging) to the APCI-MS through a flexible 1.2 m long (1 mm ID) nonheated transfer line. The experimental setup was mounted on an optical table, where the positions of the focuser, transfer line inlet, and their distance from the sample surface were precisely fixed during experiments, enabling controllable, accurate, and reproducible mass spectrum measurement of the analyzed material. All this makes our analytical system one of a kind (where an optical fiber system is combined with a transfer line system), easy to replicate, and differentiates from the above-mentioned analytical methods used for the analysis of CH materials. This easy-to-handle analytical configuration enables the direct, rapid, and accurate analysis of organic materials from the solid surfaces of artifacts under ambient conditions without requiring sample piece removal or additional surface treatment. In our previous studies, we have evaluated the performance of this LA-APCI-MS system by examining the influence of a pulsed 355 nm Nd:YAG laser impact on painting materials. We characterized the dimensions, morphology, and reproducibility of the laser-ablated craters (using optical microscopy and 3D profilometry) produced under various laser energies, pulse numbers, and incidence angles, and correlated these physical parameters with the corresponding low-resolution (LR)­MS signals.^26^ These results showed that a laser beam focused normal (perpendicular) to the surface produced the smallest crater in the analyzed materials; therefore, this geometry was used throughout the present study.

In the current study, we coupled LA with APCI-Fourier transform ion cyclotron resonance (FT-ICR)-MS. FT-ICR-MS (henceforth, high-resolution (HR)­MS) offers the highest resolution and mass-to-charge (*m*/*z*) measurement accuracy, allowing better identification of organic components in complex mixtures from the mass spectrum. The main aim of this investigation was to evaluate the effectiveness of the developed LA-APCI-HRMS system for the analysis of five handmade mock-up materials of different paint and varnish layers (copper resinate, Prussian blue oil and egg tempera paints, lead white oil paint, and matte dammar varnish) on glass and wooden plates. Additionally, an unknown material from a real-life object (the blackish-brown material from an ointment jar from a 16th-century shipwreck) was also analyzed. The obtained good-quality HRMS spectra of the analyzed materials demonstrate well the capabilities and suitability of this technique for analyzing various complex materials.

## 2 Experimental Section

### 2.1 Materials for the Analysis with LA-APCI-FT-ICR-MS

For the analysis, four mock-ups with different paint and varnish layers were prepared: two single-layered and two multilayered mock-ups (see Table 1).

**Table 1:** Description of Mock-Ups and Materials Analyzed in This Study^a^

**mock-up**		**analyzed material**
1	single-layered mock-up on glass: copper resinate	copper resinate varnish
2	single-layered mock-up on wood: Prussian blue oil paint	Prussian blue + linseed oil paint
3	3-layered mock-up on wood: matte dammar varnish, lead white oil paint, chalk primer	dammar varnish with beeswax (as a matting agent) layer
		lead white + linseed oil paint layer
4	3-layered mock-up on wood: matte dammar varnish, Prussian blue tempera paint, chalk primer	Prussian blue + egg yolk tempera paint layer

a See pictures of the mock-ups in Supporting Information (SI), Figure S1.

Copper resinate (12200), Prussian blue LUX (45202), and matte dammar varnish (UV stabilized, 79320) were purchased from Kremer Pigmente GmbH & Co. KG (Germany). Lead white pigment was synthesized at the Estonian Academy of Arts. Clarified linseed oil was from Lefranc Bourgeois (France). The hen eggs were purchased from a local grocery store. Rabbit skin glue-based chalk primer was not investigated in this study, and thus, its origin and preparation are not specified.

The preparation of the single-layered samples of copper resinate on a glass plate and Prussian blue oil paint on a wooden plate (mock-ups 1 and 2) has been described in ref 26.^26^

Mock-ups 3 and 4 were prepared in the following way: on the dried chalk primer, a paint layer was applied (in the case of mock-up 3, lead white oil paint, and in the case of mock-up 4, Prussian blue tempera paint) and left to dry for a couple of days. On top of the dried paint, matte dammar varnish (a commercial product used as-is, straight from the bottle without any modifications) was applied. Both multilayered mock-ups were left to dry at room temperature for one year before analysis.

The lead white oil paint was prepared by thoroughly mixing the pigment with linseed oil into a homogeneous paste.

Egg yolk was used to prepare Prussian blue tempera paint. At first, the egg yolk was separated from the egg white. Then, the yolk membrane was pierced with the needle, and the yolk was released into a jar. A small amount of distilled water was added and mixed into the emulsion. Dry Prussian blue pigment powder was added to the yolk-water emulsion and mixed thoroughly for viscous paint.

Additionally, the blackish-brown material from an ointment jar, found on a 16th-century shipwreck called “Nargen 1” (obtained from Estonian Maritime Museum), was analyzed using LA-APCI-HRMS (see SI, Figure S2). The blackish-brown material was taken out of the jar and brought to the lab in a resealable plastic bag. For the analysis, a piece of the material was placed on a glass plate (see chapter 2.2.1).

### 2.2 LA-APCI-FT-ICR-MS System

The developed LA-APCI-FT-ICR-MS system consists of a laser setup and transfer line that is connected through the APCI source with FT-ICR-MS (see Figure 1 and in the SI, Figure S3).

**Figure 1:**
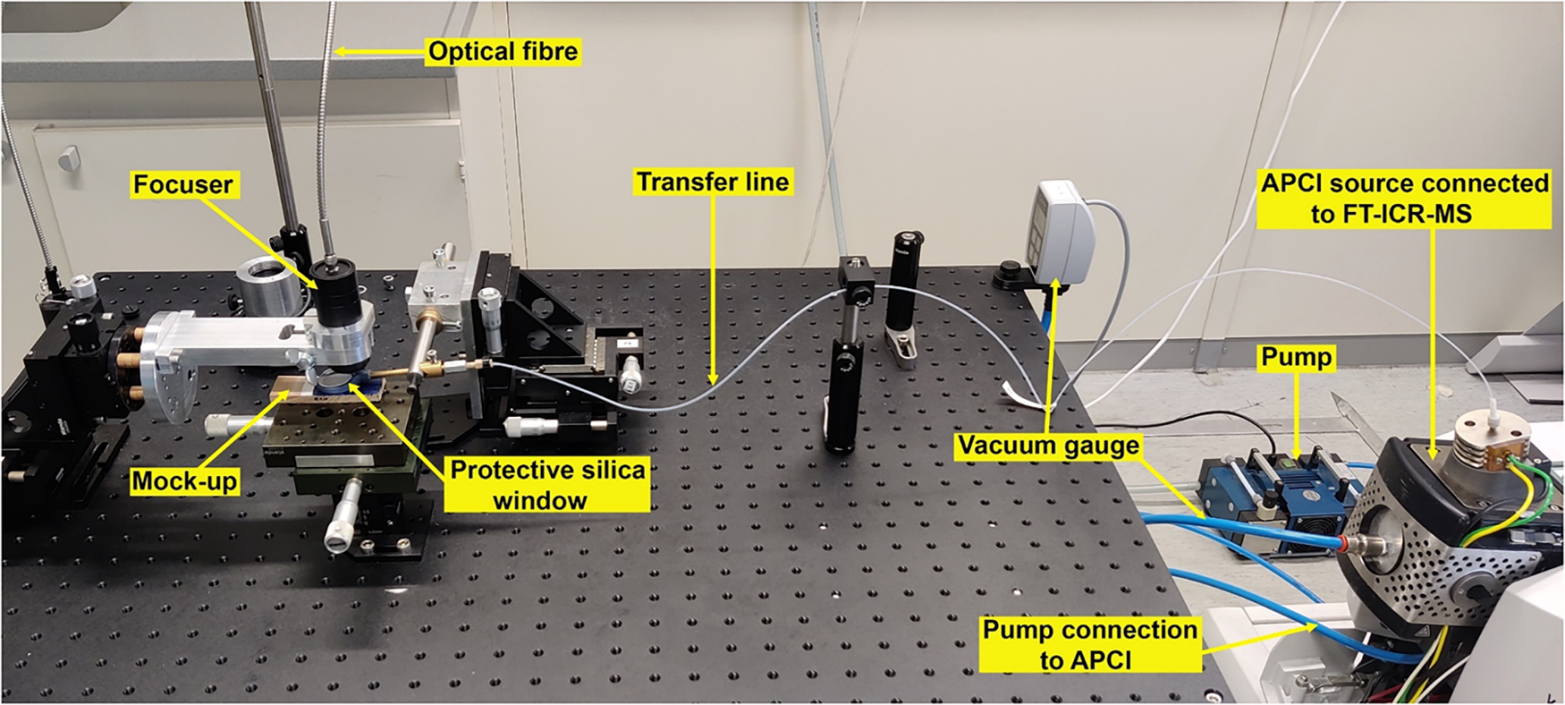
Developed LA-APCI-HRMS system.

For the ablation of materials, a compact diode-pumped Nd:YAG Q-switched laser Q1-D10-1064 from Quantum Light Instruments, Ltd. (Lithuania), coupled with a 2 m long optical UV-resistant silica fiber (Ceram Optec SIA, Latvia; numerical aperture of 0.22, core diameter of 0.8 mm) ending with a focuser unit, was utilized. In this work, the 355 nm laser output with a pulse duration of 5 ns was used. A 355 nm UV laser was used because most of the organic materials absorb light at that wavelength. The maximum laser pulse energy on the sample surface was 4.8 mJ (measured with Laserpoint A-2-D12-HPB thermal sensor connected to PLUS 2 Sensor Meter), and the corresponding energy fluence was 3.8 J/cm^2^.

The laser focuser and the 1.2 m nonheated PTFE transfer line (1 mm ID, 1.6 mm OD; BOLA, Germany) were secured to XYZ stages (Standa, Lithuania) mounted on the optical table. The focused laser beam (with a spot size of 0.4 mm in diameter at 20 mm distance from the focuser lens) was positioned perpendicularly to the horizontal sample surface and was precisely aligned using an XYZ micrometre stage, ensuring accurate and repeatable placement. For the current setup, maximum sample dimensions were limited to 200 mm × 200 mm × 20 mm (width × length × thickness). However, with minor modifications to the sample stage and optical alignment, the system could be adapted to analyze planar surfaces up to about 500 mm × 500 mm. Adjacent to the laser beam, a transfer line inlet was set at a 12° angle relative to the sample surface, and the distance of the transfer line inlet orifice to the sample surface was 1 mm. The sample-side end of the transfer line was fitted with a custom-made holder, featuring a round silica window (Thorlabs UVFS Window, diameter: 2.54 cm; thickness: 1 mm) that protected the focuser lens from evaporated particles. The transfer line holder position to the focuser unit remained fixed throughout all experiments to maintain consistency in measurements.

The transfer line was connected through the inlet of the APCI source with a Varian 910-FT-ICR mass spectrometer with a 7 T superconducting magnet. This hybrid mass spectrometry system also contains the low-resolution (LR) Varian J-320 triple quadrupole (QQQ) MS with hexapole fragmentation cell (used for experiments in our previous work^26^).

Transfer of ablated products through the transfer line was improved by slightly reducing APCI chamber pressure (absolute ∼890 mbar for copper resinate, dammar varnish, Prussian blue tempera paint, and the real-life object and ∼910 mbar for Prussian blue oil paint) using an oil-free membrane pump (type 400177, MPC 201 T, ILMVAC GmbH, Germany). The pressure gradient assisted in transporting the molecules from the ablated plume region to the APCI inlet.

Varian Omega 9.2.29 version software was used to operate the instrument and collect and process mass spectra. The software was integrated with the laser control unit, enabling synchronization of the laser and the mass spectrometer from the same computer. To avoid exceeding the laser’s pulse repetition rate of 10 Hz, the interval between laser trigger pulses was set to 101 ms. To maximize the number of ions entering the ICR cell, the ions were collected into a hexapole cell (placed before the ICR cell) that was opened 0.1 s after the first laser pulse and kept open for 1.9 s. This setup enabled the generation of 7 set laser pulses and synchronized with the ion delivery delay to the FT-ICR measurement cell. The laser pulse energy was set precisely and controlled by the laser’s control software. By starting the mass spectrum measurement in the Omega software, the laser was also automatically triggered.

#### 2.2.1 Measurements with LA-APCI-FT-ICR-MS

For the LA-APCI-FT-ICR-MS analysis, the analyzed mock-ups on a glass or wooden plate and the blackish-brown real-life sample on a glass plate were placed horizontally on the XYZ stage, and the 355 nm laser beam at a 90° angle of incidence was focused on the analyzed material surface and ablated at ambient conditions. The ablated species were transferred through the transfer line into the APCI-HRMS.

For all mock-ups, 7 laser pulses at a repetition rate of 10 Hz were used for ablation. For copper resinate on glass and dammar varnish on lead white paint, the laser pulse energy was set to 2.88 mJ, for Prussian blue oil paint, 1.92 mJ, for Prussian blue tempera paint, 0.96 mJ, and for the blackish-brown real-life sample, 3.2 mJ pulse energy was applied.

APCI-FT-ICR-MS measurements were performed in positive ion mode in the *m*/*z* range of 100–1000. The APCI corona needle current was 2 μA. The ion transfer capillary voltage was adjusted for each analyzed material: for copper resinate, 20 V; for dammar varnish, 50 V; for Prussian blue tempera paint, 30 V; for Prussian blue oil paint, 80 V. Other used APCI and FT-ICR-MS parameters are presented in SI, Table S1. Material-dependent selection of capillary voltage was optimized to avoid excessive fragmentation. For lead white oil paint, different laser and APCI-FT-ICR-MS parameters were tested, but it was not possible to acquire a good-quality mass spectrum from this material.

After the measurement of each material, the silica window and its holder, the transfer line and APCI corona needle, the MS shield, and the APCI chamber were cleaned. The silica window holder was cleaned with a cotton swab wetted with isopropanol (Honeywell CHROMASOLV, purity ≥99.9%). The silica window, corona needle, and MS shield were cleaned with a nonwoven cotton pad wetted with dichloromethane (Sigma-Aldrich Pty Ltd. LiChrosolv, purity ≥99.9%): acetonitrile (Sigma-Aldrich Pty Ltd. LiChrosolv, purity ≥99.8%): hexane (Honeywell International Inc. CHROMASOLV, purity ≥97.0%): isopropanol 1:1:1:1 (v:v:v:v) mixture (four-solvent mixture). For cleaning the transfer line, the transfer line tip was sonicated in the four-solvent mixture for 3 min, and thereafter, ∼3 mL of the same solvent mixture was injected into the transfer line with a glass syringe and finally dried with nitrogen flow.

Before the LA-APCI-FT-ICR-MS measurements, the instrument was externally calibrated with an in-house calibration solution containing 1-butyl-3-methylimidazolium and five phosphazene cations (see SI, Table S2).^27^ As a result, the *m*/*z* errors of the identified ions in the spectra were mostly below ± 2 ppm. In a few occasions, with lower intensity peaks, the errors were over ± 2 ppm. Higher *m*/*z* error values could be caused due to the instrumentation (we have a dual FT-ICR-MS device), irregular shapes of low intensity peaks, and background noise in the mass spectrum.

### 2.3 Measurements with APCI-FT-ICR-MS from Solution

For comparison, copper resinate was also analyzed from a solution with APCI-FT-ICR-MS. For this, 5 mg of the aged copper resinate sample was scraped from the glass plate and dissolved in 5 mL of the four-solvent mixture.

For measurements from solution, the same APCI-FT-ICR-MS system was used as for LA-APCI-MS experiments. The sample solution was infused with KD Scientific Inc. Gemini 88 infusion pump at a flow rate of 50 μL/min, APCI corona needle current was set to 4 μA, and the ion transfer capillary voltage was 20 V. Experiments were carried out in positive ion mode in the *m*/*z* range of 100–1000. Additional APCI and FT-ICR-MS parameters used for the analysis of copper resinate from solution can be found in SI, Table S1. The obtained mass spectrum was externally calibrated with the help of the in-house calibration solution (see SI, Table S2).

## 3 Results and Discussion

### 3.1 Comparison of Conventional APCI-FT-ICR-MS with LA-APCI-FT-ICR-MS

For comparison, aged copper resinate on a glass plate (see Table 1, mock-up 1) was analyzed using two methods: directly from the solid surface with LA-APCI-FT-ICR-MS and from a solution using conventional APCI-FT-ICR-MS. This comparison helps to better understand the performance of LA-APCI-HRMS and assess how closely its mass spectra resemble those obtained by the standard technique. Figure 2 presents mass spectra of aged copper resinate measured with LA-APCI-FT-ICR-MS from solid surface and with APCI-FT-ICR-MS from solution, and Table 2 summarizes the interpretation of these spectra (a more comprehensive interpretation of the obtained mass spectra is provided in SI, Table S3).

**Figure 2:**
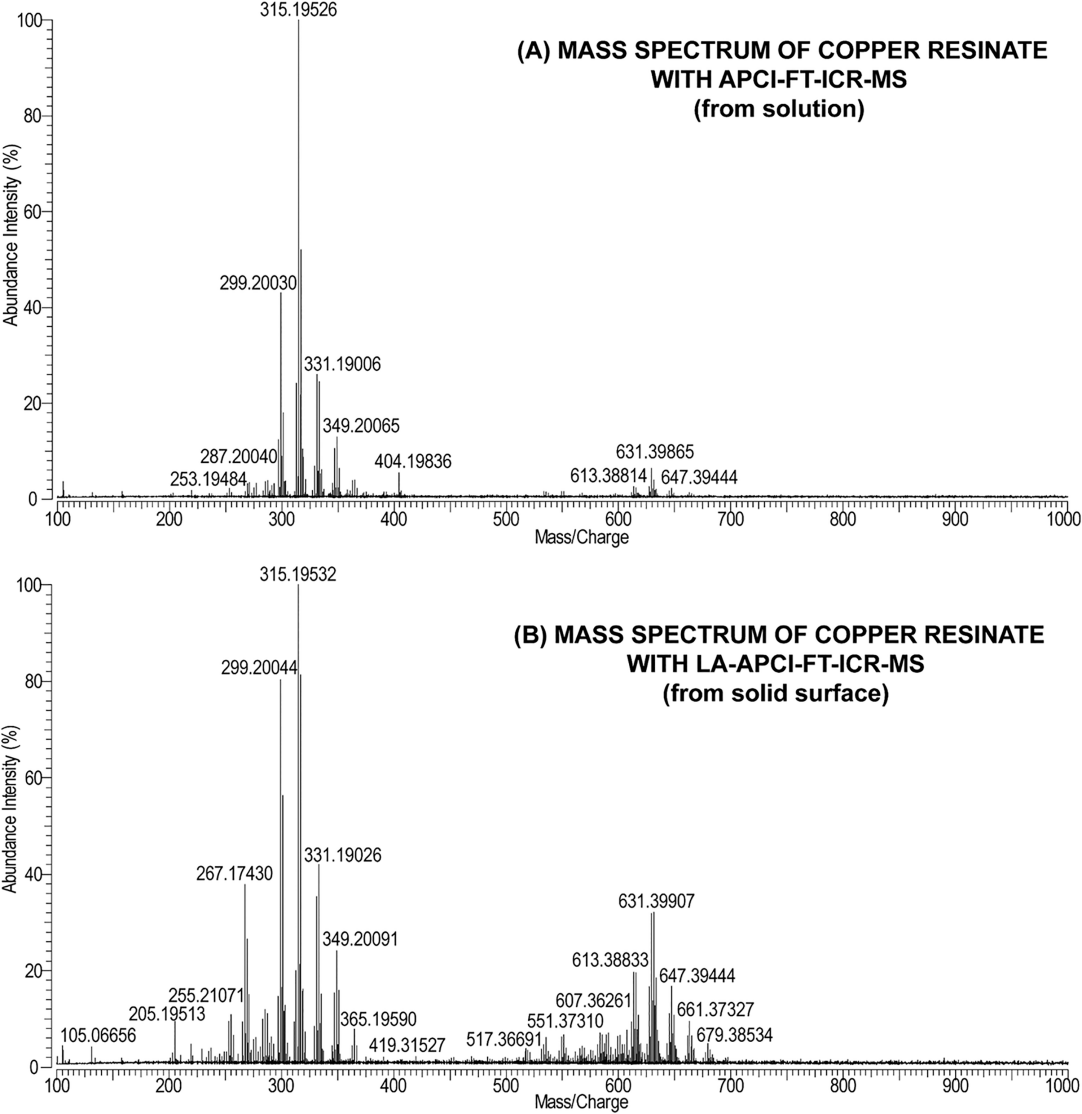
Comparison of mass spectra of aged copper resinate obtained with (A) APCI-FT-ICR-MS from solution and (B) LA-APCI-FT-ICR-MS from the solid surface.

**Table 2:** Comparison of the Interpretation Results of Mass Spectra of Aged Copper Resinate Obtained by APCI-FT-ICR-MS from Solution and LA-APCI-FT-ICR-MS from the Solid Surface^a^

APCI-FT-ICR-MS (from solution)		LA-APCI-FT-ICR-MS (from solid surface)				
measured *m/z*	Δ*m/z* (ppm)	measured *m/z*	Δ*m/z* (ppm)	Ion formulaIon		possible compound (M)
299.20030	–0.86	299.20044	–0.41	C_20_H_27_O_2_^+^	[M + H]^+^	didehydroabietic acid (C_20_H_26_O_2_)
		303.23180	–0.20	C_20_H_31_O_2_^+^	[M + H]^+^	abietic acid (C_20_H_30_O_2_)
315.19526	–0.68	315.19532	–0.48	C_20_H_27_O_3_^+^	[M + H]^+^	7-oxodehydroabietic acid (C_20_H_26_O_3_)
331.19006	–1.01	331.19026	–0.39	C_20_H_27_O_4_^+^	[M + H]^+^	15-hydroxy-7-oxodehydroabietic acid (C_20_H_26_O_4_)
631.39865	–1.06	631.39907	–0.39	C_40_H_55_O_6_^+^	[M + H]^+^	combination of 7-oxodehydroabietic acid and 15-hydroxydehydroabietic acid (C_40_H_54_O_6_)
647.39444	0.32	647.39444	0.32	C_40_H_55_O_7_^+^	[M + H]^+^	combination of 15-hydroxydehydroabietic acid and 15-hydroxy-7-oxodehydroabietic acid (C_40_H_54_O_7_)
		661.37327	–0.34	C_40_H_53_O_8_^+^	[M + H]^+^	Dimer of 15-hydroxy-7-oxodehydroabietic acid (C_40_H_52_O_8_)

a More detailed interpretation of these mass spectra is provided in SI, Table S3.

In the case of APCI-HRMS, the sample solution was directly infused into the APCI source, the evaporated molecules were then ionized by the reaction with ionic species created by the corona discharge, and the ions were separated and detected according to mass-to-charge (*m*/*z*) ratio with HRMS.^28^ In the case of LA-APCI-HRMS, at first, the copper resinate solid surface was ablated with a laser beam at ambient conditions, and the molecules from the ablated plume were transferred through the transfer line into the APCI source, where, in the gas phase, with the likely help of the H_2_O and N_2_ molecules from ambient atmosphere (instead of solvent molecules as with conventional APCI) and by the corona discharge, the sample molecules were ionized (gas-phase proton transfer) and the ions were directed to MS. It can be assumed that not all ablated molecules reached the APCI source through the transfer line, and only surviving compound ions can be detected in the mass spectra. The number of ablated molecules also depends on the laser parameters (e.g., laser pulse energy/fluence, number of laser pulses, etc.) and the analyzed material.^26^

As shown in Figure 2, the obtained LA-APCI-FT-ICR-MS spectrum is of very good quality. During the analysis, it was determined that most peaks in the mass spectrum correspond to copper resinate compounds or fragments, and the number of unassigned *m*/*z* values was low. Also, the peaks had good intensity, and the background noise did not affect the mass spectrum quality during LA-APCI-HRMS analysis.

The obtained LA-APCI-FT-ICR-MS and APCI-FT-ICR-MS mass spectra of copper resinate are comparable and contain two clusters of peaks in the *m*/*z* range of 200 to 685 (see Figure 2 and Table 2). Copper resinate consists of copper salts (mostly Verdigris — Cu­(CH_3_COO)_2_·2Cu­(OH)_2_) and colophony resin. The main components in colophony are abietic acid (C_20_H_30_O_2_) and its derivatives and aging products, e.g., dehydroabietic acid (C_20_H_28_O_2_), 15-hydroxydehydroabietic acid (C_20_H_28_O_3_), and 7-oxodehydroabietic acid (C_20_H_26_O_3_).^1^ In both mass spectra, the first cluster contains peaks of the ions of original colophony compounds and their fragments, and the second cluster belongs to the dimers and combinations of the original component ions and/or fragments formed during aging. No copper adducts of colophony compounds were detected in either mass spectra. In the obtained mass spectra, all of the ions are in protonated form ([M+H]^+^). It was observed that the first clusters in the mass spectra are quite similar, and they both contain mostly peaks corresponding to the aging products of colophony in copper resinate. For example, the most intense peak in both mass spectra belongs to 7-oxodehydroabietic acid ([C_20_H_26_O_3_+H]^+^ found at *m*/*z* 315.19532 in the LA-APCI-FT-ICR-MS spectrum and at *m*/*z* 315.19526 in the APCI-FT-ICR-MS spectrum), which is an aging product of abietic acid. At the same time, the peak corresponding to protonated abietic acid ([C_20_H_30_O_2_+H]^+^) was only present in the LA-APCI-FT-ICR-MS spectrum at *m*/*z* 303.23180 at low intensity. Aging of the copper resinate was also visible through the detection of oxidized ions (+O,+O–2H,−2H, etc.).

Most of the identified fragments were formed due to the simultaneous loss of H_2_O and CO.^29^ There were also a few fragments formed after the loss of H_2_O only. Additionally, in both mass spectra, ions of C15, C16, C17, and C18 compounds were detected that possibly correspond to fragments of the original components of copper resinate. It was noticed that peaks of these fragment ions were more intense and frequent in the LA-APCI-FT-ICR-MS spectrum. However, based on this, it cannot be concluded that the laser was more fragmenting because similar fragment peaks were also present in the mass spectrum obtained from solution (see SI, Table S3).

The most notable difference between the presented copper resinate mass spectra is the second cluster. In the case of the LA-APCI-FT-ICR-MS spectrum, the second cluster was more intense and contained significantly more peaks compared to the copper resinate mass spectrum obtained from solution. In the mass spectrum measured from solution, only dimers or combinations of abietic acid, its derivatives, and aging products were detected (C40 components). However, in the mass spectrum obtained with the laser, in addition to C40 compounds, C34–C39 components were also identified. They correspond to polymerization products of the original C20 compounds and C15–C19 fragments of copper resinate. This allows us to assume that the developed LA-APCI-HRMS technique is more suitable for monitoring the aging and polymerization processes of resinous materials compared to conventional APCI-MS.

### 3.2 Analysis of Various Materials with LA-APCI-FT-ICR-MS

To test the capabilities of the LA-APCI-FT-ICR-MS, single- and multilayered mock-ups with different materials (Prussian blue and lead white oil paints, Prussian blue tempera paint, and dammar varnish) were used for the analysis (see Table 1). To analyze binder material (linseed oil, egg yolk) in the oil paint or tempera paint with conventional chromatographic and mass spectrometric techniques, the binder must first be extracted with some solvent from the paint mixture. With LA-APCI-HRMS, the analysis can be made directly from the paint or varnish surface without additional sample preparation. That makes analysis of CH objects less damaging and easy-to-handle (no need for mechanical sampling), much quicker, and somewhat more precise.

Investigation shows that the APCI-HRMS, combined with a laser, allows controllable and precise analysis of different material layers, leaving a tiny crater (<0.4 mm in diameter) in the material layer.^26^ In the case of multilayered mock-ups (3 and 4), by adjusting laser parameters, it was possible to perform analysis of only the upper dammar varnish layer, as the laser beam did not go through to the layer below it (estimated ablation depth per pulse is between 2 and 5 μm^26^). The obtained dammar varnish mass spectra were similar, and in this study, one mass spectrum of dammar varnish on the lead white oil paint was selected.

Good-quality mass spectra could be obtained from the dark blue Prussian blue oil and tempera paints and dammar varnish, which are discussed further below (see Chapters 3.2.1–3.2.3). The obtained mass spectra of the analyzed materials are presented in Figure 3. A comprehensive interpretation of most of the *m*/*z* values of the compounds in the mass spectra of these materials is presented in SI Tables S4–S6. These mass spectra and interpretation tables provide valuable reference data for CH material studies. The results are comparable to those obtained with other solution-based techniques, e.g., with APCI-, MALDI-, ESI-MS, because the identified components are characteristic of the studied materials. As shown with the analysis of copper resinate from solid surfaces and from solution, the mass spectra are still relatively similar. A mass spectrum could not be obtained from the lead white oil paint because the paint absorbs the 355 nm UV laser very poorly. Absorbance measurements (SI, Figure S4) have confirmed that its optical absorption falls sharply above ∼330 nm, leaving too little laser energy to desorb and ablate the sample effectively. In contrast, Prussian blue oil and egg tempera paints strongly absorb at the laser wavelength,^26^ and this facilitates more efficient ablation of the material and transport to APCI-HRMS. As a result, it is possible to obtain well-defined mass spectra from these paints. The effectiveness of LA-APCI-HRMS is governed by the interplay between laser parameters and the optical properties of the analyzed material. Variations in wavelength, pulse energy, and beam profile produce markedly different ablation efficiencies across materials, directly influencing the quality of mass spectra. The experiments indicate that heterogeneous surface composition and morphology can complicate analysis, making prior knowledge of a material’s absorption spectrum, chemical composition, and thermal behavior valuable. The results indicate that pigment composition may affect paint absorption and laser ablation efficiency. Future experiments will assess pigment-dependent absorption using a 355 nm laser and investigate diverse CH materials with a Nd:YAG laser at 532 and 266 nm. This is to characterize their optical behavior, absorption coefficients, and material yield for APCI-MS analysis.

**Figure 3:**
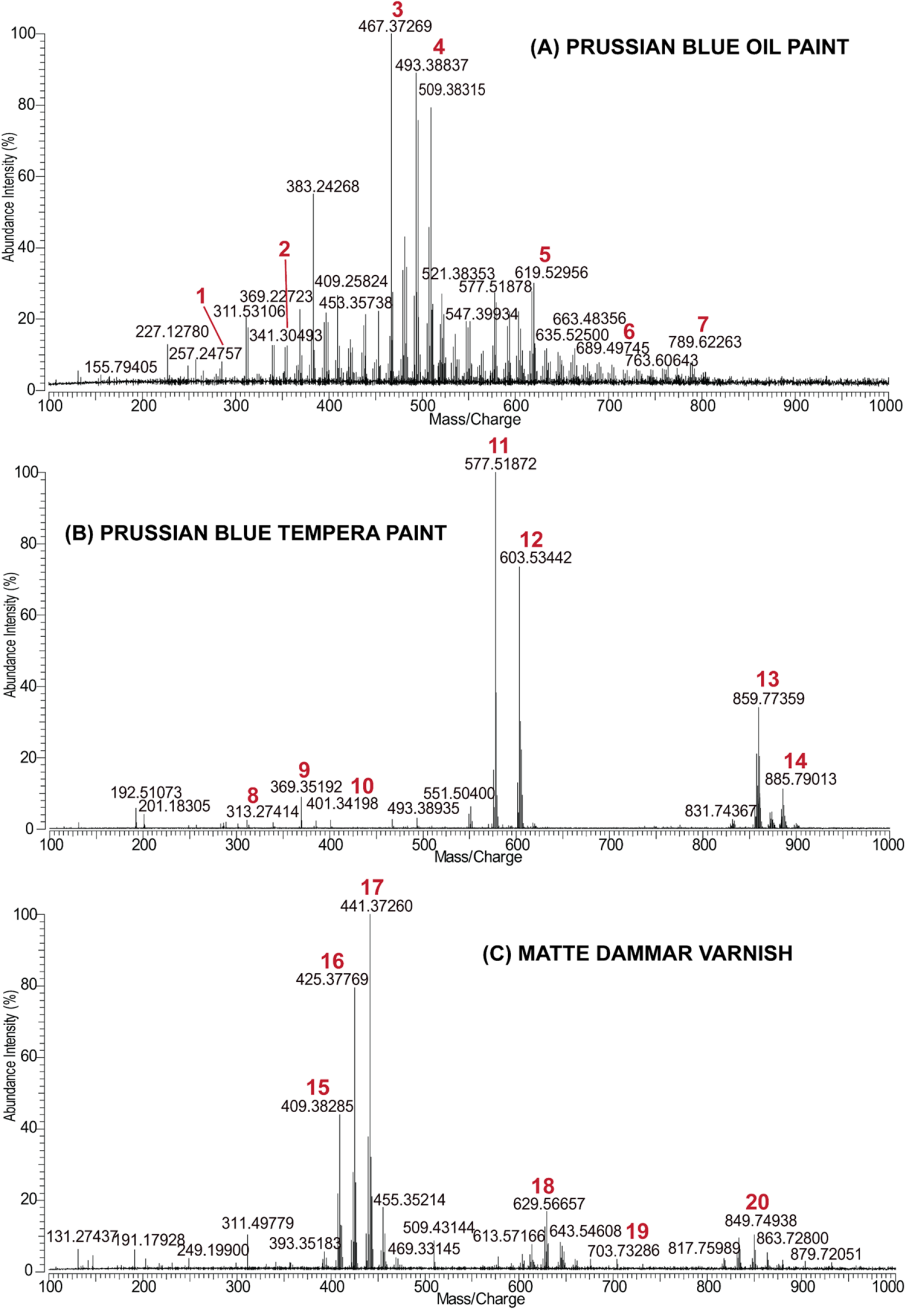
Mass spectra of (A) aged Prussian blue oil paint, (B) aged Prussian blue tempera paint, and (C) aged dammar varnish obtained with LA-APCI-FT-ICR-MS. The compounds corresponding to the peaks indicated on the figure: 1—palmitic acid, 2—glyceryl monostearate, 3—DAG may contain PoC9:1/P9-ONA, 4—DAG may contain LC9:1/LnC9:0/O9-ONA/SAz, 5—DAG may contain LnS/LO, 6—TAG may contain LSeC10:2/OSeSe, 7—TAG may contain LOAz/LnSAz, 8—glyceryl monopalmitate, 9—cholesterol, 10—3-hydroxycholest-5-en-7-one (cholesterol oxidation product), 11—DAG may contain OP/SPo, 12—DAG may contain LS/OO, 13—TAG may contain LSP/OOP/SOPo, 14—TAG may contain OOO/LSO/LnSS, 15—dammaradienol, 16 and 17—damradienone, 18—combination of hydroxydammarenone and cadinene unit, 19—monoester of triacontanol and oleic acid (from beeswax), and 20—combination of dammarenolic acid and two cadinene units. Abbreviations are written in the text (chapter 3.2.1).

#### 3.2.1 Prussian Blue Oil Paint

The mass spectrum of aged Prussian blue oil paint is presented in Figure 3. The complete detailed interpretation is given in SI, Table S4. All of the peaks in the mass spectrum belong to the components of aged linseed oil. No metal ions or adducts that belonged to the pigment were detected.

Linseed oil is a drying oil that consists mainly of mixtures of triacylglycerols (TAGs), which are composed of glycerol esterified with three fatty acids (FAs).^30,31^ In one TAG, the fatty acids are present in random combinations. The most abundant fatty acids in linseed oil are linolenic (C18:3; Ln), linoleic (C18:2; L), oleic (C18:1; O), palmitic (C16:0; P), and stearic (C18:0; S) acids.^31,32^ Besides these, it also contains minor amounts of myristic (C14:0; M), palmitoleic (C16:1; Po), arachidic (C20:0; Ar), eicosenoic (C20:1; Eic), and behenic (C22:0, Be) acids.^32,33^

In the analyzed mass spectrum, the majority of peaks in the *m*/*z* range of 365–601 belong to the diacylglycerols (DAGs), and fewer peaks in the *m*/*z* range of 313–355 belong to monoacylglycerols (MAGs) and in the *m*/*z* range of 673–789 correspond to TAGs. Also, in the mass spectrum in the *m*/*z* range of 603–665, peaks were found that made it difficult to determine whether they belonged to TAG or DAG because both compounds were possible. So, in SI Table S4, both options were presented. DAGs and MAGs are usually formed as intermediate products during the hydrolysis of TAGs in linseed oil.^1,34,35^ In the obtained mass spectrum, all of the produced ions are in protonated form ([M+H]^+^), and fragment ions (loss of nH_2_O) and oxidized (+nO) components also occur.

The interpretation of mass spectrum of aged Prussian blue oil paint showed that mostly in the glycerol backbones of DAGs and TAGs, one or more main original fatty acid(s) were substituted with some dicarboxylic acids (azelaic acid (C_9_H_16_O_4_; Az), suberic acid (C_8_H_14_O_4_; Su), sebacic acid (C_10_H_18_O_4_; Se), and pimelic acid (C_7_H_12_O_4_; Pi)) or other degradation products (decadienoic acid (C10:2), 9-oxononanoic acid (C_9_H_16_O_3_; 9-ONA), nonanoic acid (C_9_H_18_O_2_; C9:0), 2-nonenoic acid (C_9_H_16_O_2_; C9:1), 8-oxooctanoic acid (C_8_H_14_O_3_; 8-OOA), octanoic acid (C_8_H_16_O_2_; C8:0), etc.).^1,36–40^ Dicarboxylic acids and other degradation products are produced from the autoxidation of the polyunsaturated fatty acids (C18:3, C18:2, C18:1) in the linseed oil, and these are characteristic compounds of the aged linseed oil.^1,30,36^ Furthermore, it was observed that more than one TAG/DAG compound can correspond to one peak (i.e., various combinations of different fatty acids, dicarboxylic acids, or other degradation products attached to the glycerol backbone are possible) (see SI Table S4).

The intense peaks in the mass spectrum at *m*/*z* values of 467.37269, 493.38837, and 509.38315 belong to DAGs with ion formula of C_28_H_51_O_5_^+^ (may contain PoC9:1 or P9-ONA), C_30_H_53_O_5_^+^ (may contain LC9:1, LnC9:0, O9-ONA, or SAz), and C_30_H_53_O_6_^+^ (may contain L9-ONA or OAz), respectively (see SI, Table S4). In the mass spectrum, MAGs with all five main fatty acids (see SI, Table S4) can be detected: for example, glyceryl monostearate ([C_21_H_42_O_4_–H_2_O+H]^+^) can be found at *m*/*z* 341.30493 and glyceryl monolinoleate ([C_21_H_38_O_4_+H]^+^) at *m*/*z* 355.28400. Also, it can be seen that the *m*/*z* values of peaks of the TAGs in the mass spectrum were at lower *m*/*z* values (*m*/*z* below 800), probably due to the degradation (also TAG chain-scission reactions) in aged linseed oil.^41^ Usually, for fresh linseed oil, the *m*/*z* values of TAGs are in the range of 870–920.^39^ The identifiable TAG peaks were of low intensity and can be found, for example, at *m*/*z* 689.49745 (corresponds to C_41_H_69_O_8_^+^, may contain LSeC10:2 or OSeSe), at *m*/*z* 787.60811 (corresponds to C_48_H_83_O_8_^+^, may contain LLAz or OLnAz), and at *m*/*z* 789.62263 (corresponds to C_48_H_85_O_8_^+^, may contain LOAz or SLnAz). In the mass spectrum, besides DAGs, MAGs, and TAGs, peaks of some free fatty acids were also identified. In the mass spectrum, numerous components characteristic of aged linseed oil were detected. The obtained mass spectrum and interpretation table of the components in SI Table S4 serve as valuable reference data for interpreting unknown materials in the future. The results indicate that the LA-APCI-HRMS method may be somewhat fragmenting; however, not all the fragments in the mass spectrum are due to the laser ablation, as some could probably be attributed to the aging of linseed oil. The level of fragmentation of the method will be investigated in future studies.

#### 3.2.2 Prussian Blue Tempera Paint

The mass spectrum of aged Prussian blue egg yolk-containing tempera paint is presented in Figure 3 (the complete interpretation is given in SI, Table S5). All of the peaks in the mass spectrum belong only to the compounds of aged egg yolk.

The composition of egg yolk is very complex. It mainly consists of a high amount of water (48%), lipids (32–34%), proteins (16%), a smaller amount of saccharides, minerals, vitamins, and dyes.^1^ The lipids of egg yolk are mainly TAGs, phospholipids (PLs), and cholesterol.^42^

In the mass spectrum, mainly compounds of lipids were detected. In the obtained mass spectrum, all of the produced ions were in protonated form ([M+H]^+^), and fragment ions (loss of nH_2_O) and oxidized (+nO) components were also detected. In the mass spectrum, two dominant clusters of peaks can be seen in the *m*/*z* range of 547–619 (belonging to DAGs) and in the *m*/*z* range of 829–899 (belonging to TAGs). DAG peaks are almost twice as intense as the TAG peaks in the clusters. The most intense peak in the mass spectrum at *m*/*z* 577.51872 belongs to DAG that may contain fatty acid combinations like OP or SPo ([C_37_H_70_O_5_–H_2_O+H]^+^), and the second intense peak at *m*/*z* 603.53442 corresponds to DAG compound that may consist of fatty acids like LS or OO ([C_39_H_72_O_5_–H-H_2_O+H]^+^). For the TAGs, the two most intense characteristic peaks were at the *m*/*z* 859.77359 (corresponding to LSP, OOP, or SOPo ([C_55_H_102_O_6_+H]^+^)) and at *m*/*z* 885.79013 (corresponding to OOO, LSO, or LnSS ([C_57_H_104_O_6_+H]^+^)). In the mass spectrum, low-intensity MAGs and free fatty acid peaks were also detected, which were formed (like DAGs) due to the hydrolysis of TAG molecules (see SI, Table S5). There are similarities between linseed oil and the egg yolk aging process with TAGs (see Chapter 3.2.1).

In the mass spectrum, low-intensity peaks characteristic of cholesterol and its oxidation products, typically found in egg yolk, were also detected in the *m*/*z* range of 367–401. The cholesterol peak can be found at *m*/*z* 369.35192 (corresponds to [C_27_H_46_O–H_2_O+H]^+^) and one of its characteristic oxidation products, 3-hydroxycholest-5-en-7-one, at *m*/*z* 401.34198 ([C_27_H_44_O_2_+H]^+^).

In the mass spectrum obtained with the LA-APCI-FT-ICR-MS, only peaks of glycerolipids and cholesterol could be detected; peaks of phospholipids were absent. The absence of phospholipid peaks in the mass spectrum may be due to several factors. One possibility is that the laser caused excessive fragmentation, preventing sufficient intact particles from reaching the APCI source. Another possibility is that the phospholipids did not ionize effectively in APCI. This issue will be explored in more detail in future studies.

Although aged egg yolk also contains some similar glycerolipids (TAGs, DAGs, MAGs) as linseed oil, the mass spectra differ—the pattern of the clusters of peaks in the mass spectra is distinctly different (see Figure 3). The mass spectrum of aged Prussian blue tempera paint can also be used as a reference mass spectrum for the analysis of unknown binder materials in painted artifacts.

#### 3.2.3 Dammar Varnish

The mass spectrum of aged matte dammar varnish obtained with LA-APCI-FT-ICR-MS is presented in Figure 3. The detailed interpretation of this mass spectrum can be found in SI, Table S6. The analyzed matte dammar varnish contains primarily dammar resin, with bleached beeswax used as a mattifying additive. Dammar resin consists of tetra- and pentacyclic triterpenoids, and its polymeric fraction is based on sesquiterpene cadinene (C_15_H_24_).^43^ Beeswax contains long-chain hydrocarbons, e.g., heptacosane (C_27_H_56_) and pentacosane (C_25_H_52_), and long-chain alcohols and carboxylic acids and their combinations (mono-, di-, triesters), for example, triacontanyl palmitate (C_46_H_92_O_2_, monoester of triacontanol (C_30_H_62_O) and palmitic acid (C_16_H_32_O_2_)).^38^

The mass spectrum of matte dammar varnish contains three clusters of peaks (*m*/*z* range, 390–880). All detected ions were formed by protonation, and fragmentation was detected to a lesser extent compared to copper resinate. Mostly fragments formed by the loss of H_2_O were observed, but some fragments with the simultaneous loss of H_2_O and CO were also identified (see SI, Table S6).

In the first cluster (*m*/*z* 390–475), the most intense peaks belong to triterpenoid components characteristic of dammar resin. For example, the peak at *m*/*z* 441.37260 was assigned to dammaradienone ([C_30_H_48_O+O+H]^+^), the peak at *m*/*z* 425.37769 to hydroxydammarenone ([C_30_H_50_O_2_–H_2_O+H]^+^), the peak at *m*/*z* 409.38285 to dammaradienol ([C_30_H_50_O-H_2_O+H]^+^), and the peak at *m*/*z* 455.35214 to oleanonic/ursonic acid ([C_30_H_46_O_3_+H]^+^). In addition, protonated nor-amyrone ([C_29_H_46_O +H]^+^) at *m*/*z* 411.36232 and its oxidation products at *m*/*z* 425.34166 ([C_29_H_46_O–2H+O+H]^+^) and *m*/*z* 441.33682 ([C_29_H_46_O–2H+2O+H]^+^) were also identified in the mass spectrum (see more in SI, Table S6). The second and third clusters (in the *m*/*z* ranges of 610–665 and 815–880) contain peaks corresponding to the combinations of dammar resin components with one or two cadinene units, respectively. For example, the peak at *m*/*z* 629.56657 was identified as C_45_H_73_O^+^ (combination of hydroxydammarenone and cadinene) and the peak at *m*/*z* 849.74938 as C_60_H_97_O_2_^+^ (combination of dammarenolic acid and 2 cadinene units).

In the mass spectrum, a few low-intensity peaks belonging to the compounds of beeswax were also detected. For example, the monoester of triacontanol and oleic acid at *m*/*z* 703.73286 (corresponding to [C_48_H_94_O_2_+H]^+^) and the diester of triacontane-1,30-diol with palmitic acid and myristic acid at *m*/*z* 903.90853 ([C_60_H_118_O_4_+H]^+^) (see more in SI, Table S6).

The results demonstrated that with the developed LA-APCI-HRMS system, it is possible to obtain a mass spectrum from the upper layer′s material without significant interference or dominating peaks from the underlying material. The study also indicated that the system is very suitable for the analysis of natural resin-based varnishes, such as dammar varnish and copper resinate, as it was easy to obtain good-quality and reproducible mass spectra from these thin, brittle, and shiny aged complex materials.

### 3.3 Analysis of Real-Life Sample with LA-APCI-FT-ICR-MS

The investigation with LA-APCI-FT-ICR-MS was also performed directly on the real-life sample piece obtained from a small (approximately 5 cm tall) cylindrical jar that was found on a 16th-century shipwreck named “Nargen 1.” The shipwreck was discovered in the summer of 2015 near the island of Naisaar (Nargen) in Tallinn Bay (Baltic Sea, Northern Estonia).^44^ The analyzed blackish-brown material was assumed to be an Early Modern Age medicine (ointment) because other finds on the ship’s wreckage indicated the cargo of an apothecary.^44^

The mass spectrum of the analyzed blackish-brown material is presented in Figure 4, and a more detailed interpretation of this mass spectrum is provided in SI, Table S7.

**Figure 4:**
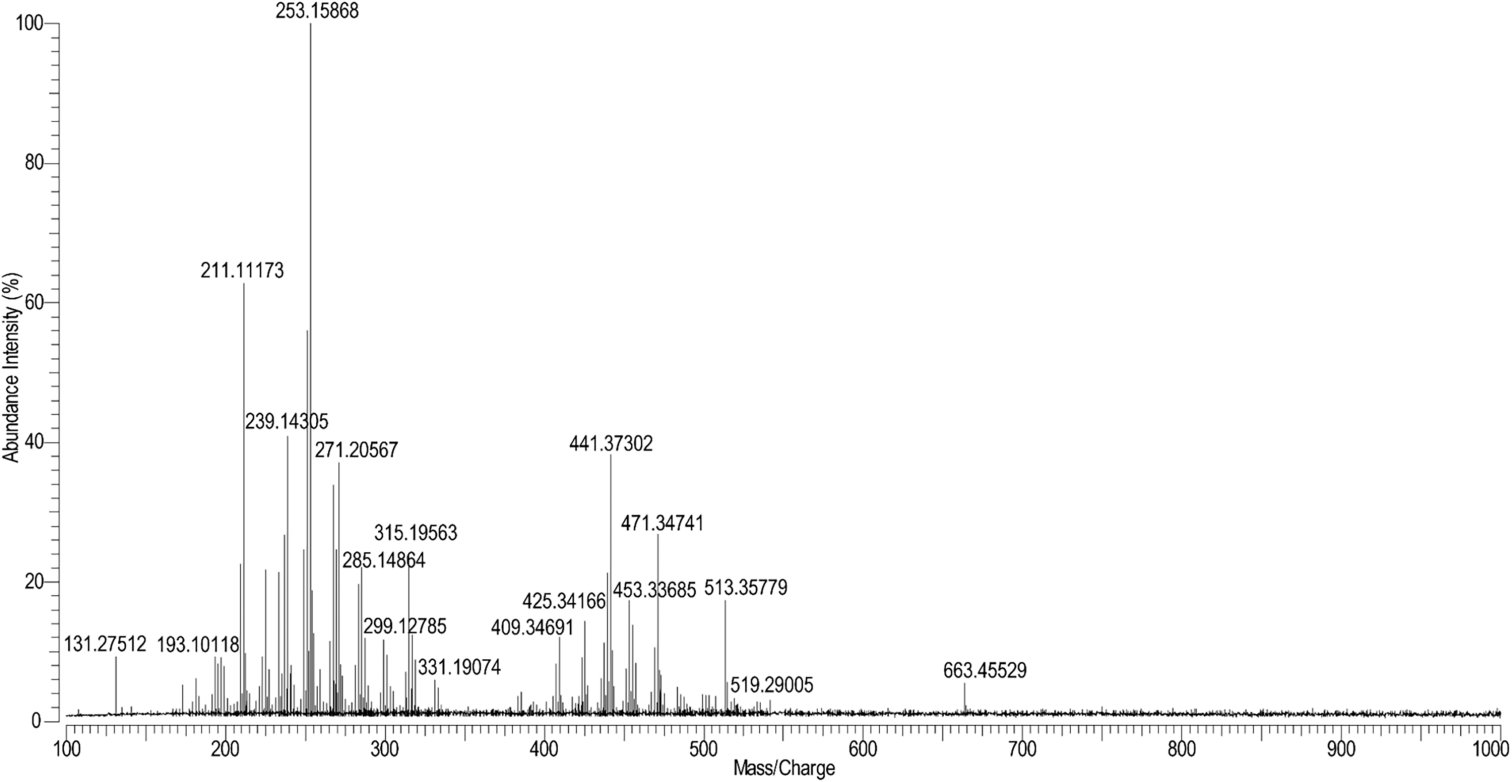
Mass spectrum of blackish-brown material from an ointment jar from a 16th-century shipwreck obtained with LA-APCI-FT-ICR-MS.

The mass spectrum in Figure 4 displays two clusters of peaks. In the first cluster (*m*/*z* range: 170–335), several *m*/*z* values corresponded to the components of pine tar. Pine tar is obtained by pyrolytic heating of pine wood. Its composition is complex and, in addition to the pine resin (and also colophony) components (abietic acid, its aging products, etc.), it also contains numerous low molecular weight (generally below 300 Da) hydrocarbons such as retene and simonellite (considered as pine tar markers).^45,46^ In the mass spectrum, for example, it was possible to identify 7-oxodehydroabietic acid at *m*/*z* 315.19563 (corresponding to [C_20_H_26_O_3_+H]^+^) and 299.20077 ([C_20_H_26_O_2_+H]^+^) and 15-hydroxydehydroabietic acid at *m*/*z* 271.20567 ([C_20_H_28_O_3_–H_2_O–CO+H]^+^). Additionally, low-intensity peaks belonging to retene at *m*/*z* 235.14833 ([C_18_H_18_+H]^+^) and simonellite at *m*/*z* 253.19523 ([C_19_H_24_+H]^+^) were detected, confirming the presence of pine tar in the sample (see SI, Figure S5). In the second cluster (*m*/*z* range: 380–540), many peaks corresponding to triterpenoids and their fragments were identified, e.g., the peak at *m*/*z* 441.37302 was assigned as C_30_H_49_O_2_^+^, the peak at *m*/*z* 453.33685 as C_30_H_45_O_3_^+^, and the peak at *m*/*z* 471.34741 as C_30_H_47_O_4_^+^. However, it is not possible to determine a specific material based on these compounds. There are several materials with similar triterpenoid composition, such as mastic resin, myrrh, birch bark tar, etc.^38^ In addition to pine tar and triterpenoid compounds, peaks corresponding to some unknown material(s) (mainly in the *m*/*z* range of 480–665) were detected in the mass spectrum (see SI, Table S7). The interpretation of unknown materials requires further investigation in the future.

The analysis of the blackish-brown sample showed that the LA-APCI-HRMS system can be successfully used for analysis of this complex, dark, glossy, brittle material that has aged in seawater for centuries. The laser ablated only a tiny amount of material and left barely visible laser marks on the analyzed sample surface (see SI, Figure S2). The ablated amount of material was still enough for obtaining a good mass spectrum where marker peaks characteristic of pine tar (e.g., retene) could be identified. Pine tar and pine resin can be difficult to distinguish in analysis because their compositions are very similar, and the few marker components of pine tar may not be recovered during insufficient sample preparation (e.g., incomplete dissolution).

## 4 Conclusions

The main aim of this study was to investigate the capabilities of the developed LA-APCI-HRMS original system for analyzing under ambient conditions five aged mock-up materials (copper resinate, Prussian blue oil and egg tempera paints, lead white oil paint, and matte dammar varnish) and real-life objects’ material.

The first obtained results show the potential of this technique. From the five analyzed mock-up materials, four materials (copper resinate, dammar varnish, and Prussian blue oil and egg tempera paints) gave good-quality and identifiable mass spectra. The obtained mass spectra had characteristic component peaks with high mass accuracy (majority < ± 1 ppm, some up to ± 2 ppm, in a few cases over ± 2 ppm). The mass spectra contained characteristic compound peaks of the materials in the mass range of *m*/*z* 100 to 910, indicating that the laser can also ablate larger molecules with *m*/*z* values above 700, which is beneficial in analyzing aged and polymerized cultural heritage materials. In the mass spectra of the analyzed materials, some fragmentation of the components was detected. Whether this fragmentation arises from material aging or from the combined effects of laser ablation and APCI remains uncertain. Future work will more thoroughly investigate fragmentation behavior using the LA-APCI-HRMS technique.

The mass spectrum from the lead white oil paint could not be obtained. This was due to the poor absorption of the 355 nm laser wavelength in the lead white oil paint. The analysis results and the quality of the mass spectrum strongly depend on the material’s optical properties (specifically, its absorption at the laser wavelength) and its chemical composition. In future studies, a wide range of materials (e.g., different pigment and binder paint mixtures, varnishes, etc.) varying in color tone, layer thickness, gloss or matt finish, and chemical composition will be systematically investigated to comprehensively evaluate the capabilities of the developed LA-APCI-HRMS system. In addition to the 355 nm laser, 266 and 532 nm lasers will also be tested to expand the capabilities of the developed analytical system.

In this study, using copper resinate as an example, the mass spectrum obtained with LA-APCI-HRMS was compared to that obtained with APCI-HRMS from a solution. The mass spectra were quite similar, containing the same compounds of copper resinate. The biggest difference was in the second cluster with compounds of higher *m*/*z* values, where for LA-APCI-HRMS, this cluster contained significantly more intense peaks. It was noticed that with LA-APCI-HRMS, it was possible to detect more oxidized, polymerized products and fragment ions of copper resinate. These analyses confirm that mass spectra obtained with LA-APCI-HRMS do not largely differ from mass spectra obtained with APCI-HRMS and would be comparable with those obtained with other ion sources and mass spectrometric devices (like ESI-MS, MALDI-MS, etc.).

The applicability of the LA-APCI-HRMS method was also tested on a sample from a 16th-century shipwreck. Based on the obtained characteristic mass spectrum, pine tar was detected. The results of this study demonstrated that the elaborated analytical system is capable of successfully analyzing the chemical composition of artifacts that are several hundred years old.

This article introduced a developed laser ablation-based APCI-FT-ICR-MS system and demonstrated its capability of analyzing selected types of materials directly on the solid surface under ambient conditions without any sample preparation. The developed LA-APCI-HRMS system expands the possibilities for investigating cultural heritage objects in a simpler and easier way, providing valuable mass spectrometric information about the components of the materials without causing significant surface damage to the artifacts. The developed LA-APCI-HRMS design (combination of flexible optical fiber and transfer line systems) is one of a kind and has potential for the future to become a portable, easy-to-handle setup. Additionally, the LA-APCI-HRMS technique is not limited to the analysis of cultural heritage materials and can be applied to various research fields (e.g., materials science, forensics, physics, etc.).

## Supplementary Material



## Notes

Fully interpreted mass spectra of analyzed materials obtained with LA-APCI-HRMS, images of analyzed mock-up materials and real-life objects, an image of the developed LA-APCI-FT-ICR-MS system, tables of APCI and FT-ICR-MS parameters and used calibration standards for external calibration, and spectrum of extinction coefficient of lead white oil paint (PDF)

## References

[1] Organic Mass Spectrometry in Art and Archaeology; Colombini, M. P. ; Modugno, F. , Eds.; Wiley: Chichester, West Sussex, 2009.

[2] Hoffmann, E. de. ; Stroobant, V. Mass Spectrometry: Principles and Applications, 3rd ed.; J. Wiley: Chichester, West Sussex, England ; Hoboken, NJ, 2007.

[3] Geddes
da Filicaia E., Evershed R. P., Peggie D. A. (2023). Review of Recent
Advances on the Use of Mass Spectrometry Techniques for the Study
of Organic Materials in Painted Artworks. Anal.
Chim. Acta.

[4] Degano I., La Nasa J. (2016). Trends in High Performance
Liquid Chromatography for
Cultural Heritage. Top. Curr. Chem..

[5] Rigante E. C. L., Calvano C. D., Ventura G., Cataldi T. R. I. (2025). Look but Don’t
Touch: Non-Invasive Chemical Analysis of Organic Paint Binders –
A Review. Anal. Chim. Acta.

[6] Rankin-Turner S., Sears P., Heaney L. M. (2023). Applications of Ambient Ionization
Mass Spectrometry in 2022: An Annual Review. Anal. Sci. Adv..

[7] Kuo T.-H., Dutkiewicz E. P., Pei J., Hsu C.-C. (2020). Ambient Ionization
Mass Spectrometry Today and Tomorrow: Embracing Challenges and Opportunities. Anal. Chem..

[8] Vettorazzo C., Sandström E., Troalen L. G., Mackay C. L., Hulme A. N. (2025). Heritage
Science Applications of Ambient Mass Spectrometry. Anal. Methods.

[9] Alvarez-Martin A., Cleland T. P., Kavich G. M., Janssens K., Newsome G. A. (2019). Rapid Evaluation
of the Debromination Mechanism of Eosin in Oil Paint by Direct Analysis
in Real Time and Direct Infusion-Electrospray Ionization Mass Spectrometry. Anal. Chem..

[10] Sandström E., Vettorazzo C., Mackay C. L., Troalen L. G., Hulme A. N. (2023). Development
and Application of Desorption Electrospray Ionization Mass Spectrometry
for Historical Dye Analysis. Anal. Chem..

[11] Aguilar-Rodríguez P., Mejía-González A., Zetina S., Colin-Molina A., Rodríguez-Molina B., Esturau-Escofet N. (2021). Unexpected
Behavior of Commercial Artists’ Acrylic Paints under UVA Artificial
Aging. Microchem. J..

[12] Watts K. E., Lagalante A. F. (2018). Method Development for Binding Media
Analysis in Painting
Cross-Sections by Desorption Electrospray Ionization Mass Spectrometry. Rapid Commun. Mass Spectrom..

[13] Newsome G. A., Kayama I., Brogdon-Grantham S. A. (2018). Direct Analysis in Real Time Mass
Spectrometry (DART-MS) of Discrete Sample Areas without Heat Damage. Anal. Methods.

[14] Alvarez-Martin A., Quanico J., Scovacricchi T., Avranovich Clerici E., Baggerman G., Janssens K. (2023). Chemical Mapping of the Degradation
of Geranium Lake in Paint Cross Sections by MALDI-MSI. Anal. Chem..

[15] Newsome G. A., Martin K. M. (2023). Non-Proximate Sampling and Photoionization
for Damage-Free
Mass Spectrometric Analysis of Intact Native American Baskets. Anal. Chem..

[16] Cleland T. P., Newsome G. A., Hollinger R. E. (2019). Proteomic
and Direct Analysis in
Real Time Mass Spectrometry Analysis of a Native American Ceremonial
Hat. Analyst.

[17] Ahn K., Schedl A., Zweckmair T., Rosenau T., Potthast A. (2018). Fire-Induced
Structural Changes and Long-Term Stability of Burned Historical Rag
Papers. Sci. Rep..

[18] Vahur S., Treshchalov A., Lohmus R., Teearu A., Niman K., Hiiop H., Kikas J., Leito I. (2024). Laser-Based
Analytical
Techniques in Cultural Heritage Science – Tutorial Review. Anal. Chim. Acta.

[19] Cheng S.-C., Shiea C., Huang Y.-L., Wang C.-H., Cho Y.-T., Shiea J. (2017). Laser-Based Ambient
Mass Spectrometry. Anal.
Methods.

[20] Herdering C., Reifschneider O., Wehe C. A., Sperling M., Karst U. (2013). Ambient Molecular
Imaging by Laser Ablation Atmospheric Pressure Chemical Ionization
Mass Spectrometry. Rapid Commun. Mass Spectrom..

[21] Hirotani R., Miyoshi Y., Sendilraj V., Hazama H. (2024). Atmospheric Pressure
Mass Spectrometry Imaging Using Electrospray-Assisted Laser Desorption/Ionization
with Gas Transportation through a Heated Tube and Minimal Sample Preparation. Mass Spectrom..

[22] Compton L. R., Reschke B., Friend J., Powell M., Vertes A. (2015). Remote Laser
Ablation Electrospray Ionization Mass Spectrometry for Non-Proximate
Analysis of Biological Tissues. Rapid Commun.
Mass Spectrom..

[23] Simon D., Horkovics-Kováts G. S., Xiang Y., Battle R. A., Wang Y., Abda J., Papanastasiou D., Stavrakaki S. M., Ho H.-Y., Wang H., Schäffer R., Karancsi T., Mroz A., Pap I., Lagache L., Balog J., Fournier I., Murray R. T., Bunch J., Takáts Z. (2025). Subcellular-Resolution Molecular
Pathology by Laser
Ablation–Rapid Evaporative Ionization Mass Spectrometry. Anal. Chem..

[24] Stopka S. A., Khattar R., Agtuca B. J., Anderton C. R., Paša-Tolić L., Stacey G., Vertes A. (2018). Metabolic
Noise and Distinct Subpopulations
Observed by Single Cell LAESI Mass Spectrometry of Plant Cells in
Situ. Front. Plant Sci..

[25] Shrestha B., Vertes A. (2009). In Situ Metabolic Profiling of Single Cells by Laser
Ablation Electrospray Ionization Mass Spectrometry. Anal. Chem..

[26] Vahur S., Teearu A., Lohmus R., Leissoo M., Treshchalov A., Lungevics J., Arju G., Hiiop H. (2025). Characterisation of
Laser-Ablated Craters of Different Painting Materials and Evaluation
with Modified LA-APCI-MS System. Talanta.

[27] Teearu A., Vahur S., Rodima T., Herodes K., Bonrath W., Netscher T., Tshepelevitsh S., Trummal A., Lõkov M., Leito I. (2017). Method Development
for the Analysis of Resinous Materials with MALDI-FT-ICR-MS:
Novel Internal Standards and a New Matrix Material for Negative Ion
Mode. J. Mass Spectrom..

[28] Gross, J. H. Mass Spectrometry: A Textbook, 3rd ed.; Springer International Publishing : Imprint: Springer: Cham, 2017 10.1007/978-3-319-54398-7.

[29] van den Berg, K. J. Analysis of Diterpenoid Resins and Polymers in Paint Media and Varnishes : With an Atlas of Mass Spectra, MOLART report 10; FOM Institute AMOLF: Amsterdam, Netherlands; 2003.

[30] Degano I., La Nasa J., Ghelardi E., Modugno F., Colombini M. P. (2016). Model Study
of Modern Oil-Based Paint Media by Triacylglycerol Profiling in Positive
and Negative Ionization Modes. Talanta.

[31] Tammekivi E., Vahur S., Kekišev O., Werf I. D. van. der., Toom L., Herodes K., Leito I. (2019). Comparison
of Derivatization
Methods for the Quantitative Gas Chromatographic Analysis of Oils. Anal. Methods.

[32] Lazzari M., Chiantore O. (1999). Drying and
Oxidative Degradation of Linseed Oil. Polym.
Degrad. Stab..

[33] Danish M., Nizami M. (2019). Complete Fatty Acid
Analysis Data of Flaxseed Oil Using
GC-FID Method. Data Brief.

[34] van
Dam E. P., van den Berg K. J., Proaño Gaibor A. N., van Bommel M. (2017). Analysis of Triglyceride Degradation Products in Drying
Oils and Oil Paints Using LC–ESI-MS. Int. J. Mass Spectrom..

[35] Erhardt D., Tumosa, Charles S., Mecklenburg M. F. (2000). Natural
and Accelerated Thermal Aging of Oil Paint
Films. Stud. Conserv..

[36] Tammekivi E., Vahur S., Vilbaste M., Leito I. (2021). Quantitative GC–MS
Analysis of Artificially Aged Paints with Variable Pigment and Linseed
Oil Ratios. Molecules.

[37] Tirat S., Degano I., Echard J.-P., Lattuati-Derieux A., Lluveras-Tenorio A., Marie A., Serfaty S., Le Huerou J.-Y. (2016). Historical
Linseed Oil/Colophony Varnishes Formulations: Study of Their Molecular
Composition with Micro-Chemical Chromatographic Techniques. Microchem. J..

[38] Mills, J. S. ; White, R. The Organic Chemistry of Museum Objects, 2nd ed.; Butterworth-Heinemann series in conservation and museology; Butterworth-Heinemann: Oxford, 2003.

[39] Wei S., Schreiner M., Rosenberg E., Guo H., Ma Q. (2011). The Identification
of Binding Media in the Tang Dynasty Chinese Wall Paintings by Using
Py-GC/MS and GC/MS Techniques. Int. J. Conserv.
Sci..

[40] van
den Berg J. D. J., van den Berg K. J., Boon J. J. (2002). Identification of
Non-Cross-Linked Compounds in Methanolic Extracts of Cured and Aged
Linseed Oil-Based Paint Films Using Gas Chromatography–Mass
Spectrometry. J. Chromatogr. A.

[41] Calvano C. D., van der Werf I. D., Palmisano F., Sabbatini L. (2011). Fingerprinting
of Egg and Oil Binders in Painted Artworks by Matrix-Assisted Laser
Desorption Ionization Time-of-Flight Mass Spectrometry Analysis of
Lipid Oxidation by-Products. Anal. Bioanal.
Chem..

[42] Teuber K., Schiller J., Fuchs B., Karas M., Jaskolla T. W. (2010). Significant
Sensitivity Improvements by Matrix Optimization: A MALDI-TOF Mass
Spectrometric Study of Lipids from Hen Egg Yolk. Chem. Phys. Lipids.

[43] Vahur S., Teearu A., Haljasorg T., Burk P., Leito I., Kaljurand I. (2012). Analysis of Dammar Resin with MALDI-FT-ICR-MS and APCI-FT-ICR-MS. J. Mass Spectrom..

[44] Mäss V., Russow E. (2015). A Delivery for a Pharmacy? Exceptional Collection of
Early Modern Age Finds from the Sea Bed of the Tallinn Bay: Saadetis
Apteekrile? Erandlik Kogum Varauusaegseid Leide Tallinna Lahe Põhjast. Arheoloogilised Välitööd Eestis.

[45] Salvini L., Pecci A., Giorgi G. (2008). Cooking Activities
during the Middle
Ages: Organic Residues in Ceramic Vessels from the Sant’Antimo
Church (Piombino–Central Italy). J. Mass
Spectrom..

[46] Davara J., Jambrina-Enríquez M., Rodríguez de Vera C., Herrera-Herrera A. V., Mallol C. (2023). Pyrotechnology and Lipid Biomarker
Variability in Pine Tar Production. Archaeol.
Anthropol. Sci..

