# Supplementary material for: Laser Ablation APCI-HRMS Method for the Analysis of Cultural Heritage Materials
Source: J Am Soc Mass Spectrom. 2025 Dec 18;37(1):226–37. doi: 10.1021/jasms.5c00308 (PMC12784387; doi:10.1021/jasms.5c00308)
Supplement: Supplementary file 1 [file js5c00308_si_001.pdf]

Supporting Information

to the article

Laser ablation APCI-HRMS method for the analysis of cultural heritage materials

Anu Teearu[a], Martin Leissoo[a], Rynno Lohmus[b], Alexey Treshchalov[b], Tõiv Haljasorg[a], Victor Augusto Xavier da Silveira[c], Hilkka Hiiop[a,d], Signe Vahur[a,*]

[a]Institute of Chemistry, University of Tartu, Ravila 14A, 50411, Tartu, Estonia
[b]Institute of Physics, University of Tartu, W. Ostwaldi 1, 50411, Tartu, Estonia
[c]Agilent Technologies Deutschland GmbH, Hewlett-Packard-Str. 8, D-76337, Waldbronn, Germany
[d]Department of Cultural Heritage and Conservation, Estonian Academy of Arts, Põhja pst 7, 10412, Tallinn, Estonia
*Corresponding author: e-mail signe.vahur@ut.ee.

Contents

Figure S1. Mock-ups on glass and wooden plates analysed in this study S-2

Figure S2. Blackish brown material from an ointment jar, found on a 16th-century shipwreck called "Nargen 1" S-2

Figure S3. Developed LA-APCI-FT-ICR-MS system S-3

Table S1. Additional parameters used for obtaining mass spectra with LA-APCI-FT-ICR-MS from solid surface and APCI-FT-ICR-MS from solution S-3

Table S2. Calibration standards used for external calibration in LA-APCI-FT-ICR-MS and APCI-FT-ICR-MS analysis S-4

Table S3. Interpretation of mass spectra of aged copper resinate obtained with APCI-FT-ICR-MS from solution and LA-APCI-FT-ICR-MS from solid surface. S-5

Table S4. Interpretation of the mass spectrum of Prussian blue linseed oil paint obtained with LA-APCI-FT-ICR-MS. S-10

Table S5. Interpretation of the mass spectrum of Prussian blue tempera (egg yolk) paint obtained with LA-APCI-FT-ICR-MS S-13

Table S6. Interpretation of mass spectrum of matte dammar varnish obtained with LA-APCI-FT-ICR-MS S-15

Figure S4. The spectrum of extinction coefficient of lead white oil paint S-17

Figure S5. Mass spectrum of blackish brown material from an ointment jar from a 16th-century shipwreck obtained with LA-APCI-FT-ICR-MS. Zoomed are peaks of retene and simonellite (highlighted in red), which are marker compounds of pine tar. S-18

Table S7. Interpretation of mass spectrum obtained from blackish brown material from an ointment jar from a 16th-century shipwreck with LA-APCI-FT-ICR-MS. S-18

References S-22

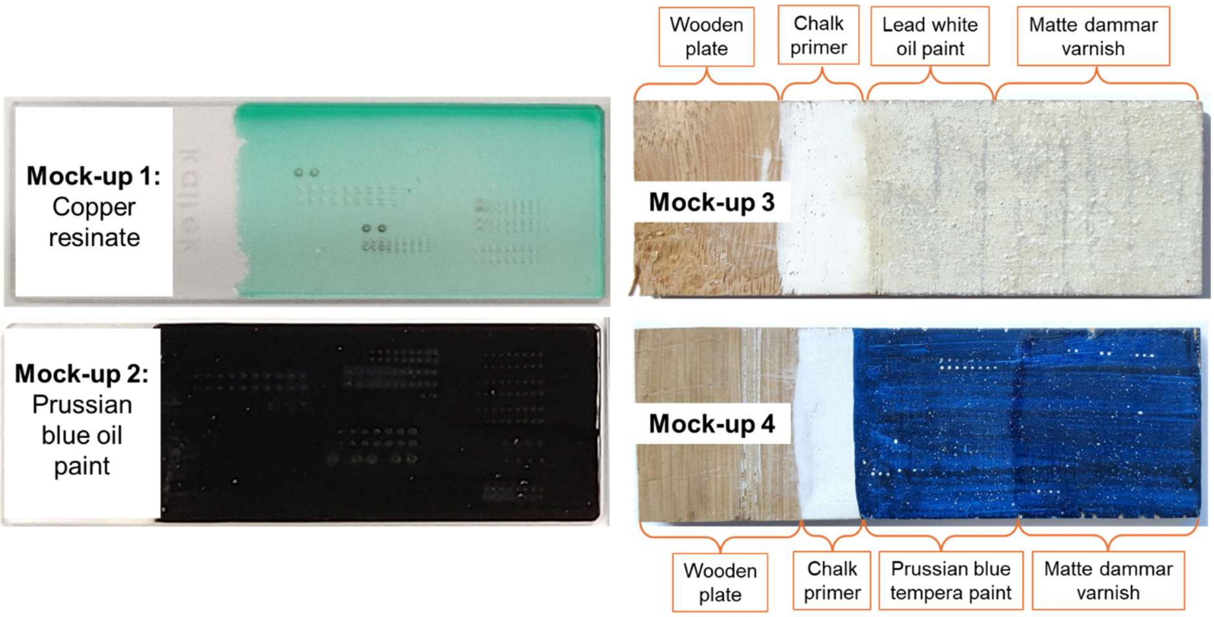

Figure S1. Mock-ups on glass and wooden plates analysed in this study.

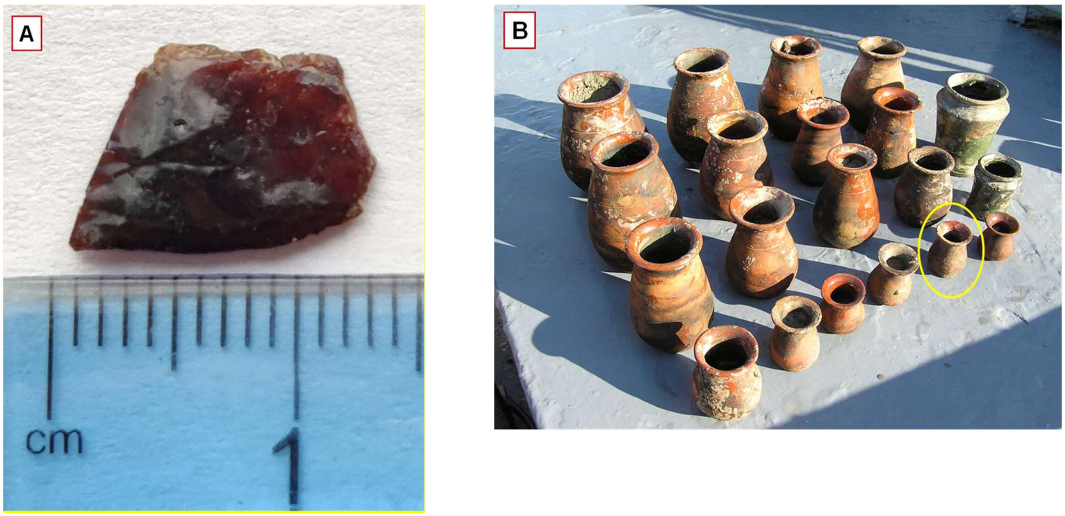

Figure S2. Blackish brown material from an ointment jar, found on a 16th-century shipwreck called "Nargen 1". (A) Blackish brown material piece analysed with LA-APCI-FT-ICR-MS. (B) The collection of ointment jars from a 16th-century shipwreck (photo: Estonian Maritime Museum, V. Mäss). The blackish brown material was obtained from the small jar highlighted with yellow circle.

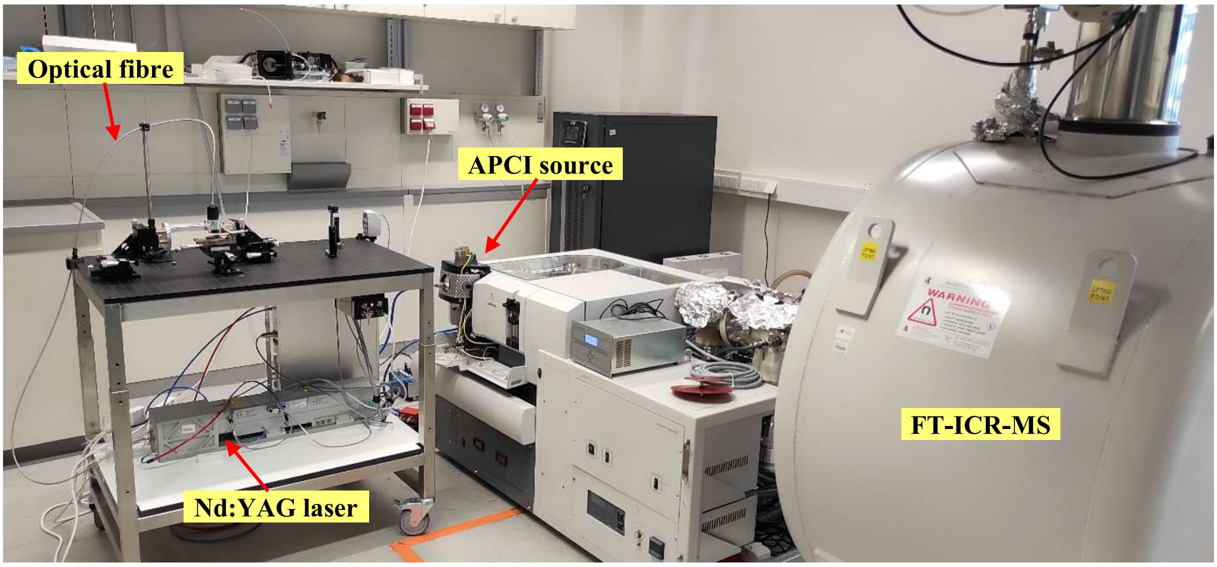

Figure S3. Developed LA-APCI-FT-ICR-MS system.

Table S1. Additional parameters used for obtaining mass spectra with LA-APCI-FT-ICR-MS from solid surface and APCI-FT-ICR-MS from solution. More important parameters are described in the main text.

Parameter	LA-APCI-FT-ICR-MS (from solid surface)	APCI-FT-ICR-MS (from solution)
**APCI parameters (all materials):**		
Flow rate (µl/min)	-	50
APCI spray shield voltage (V)	600	600
APCI drying gas (N_2)	12 psi (830 mbar) at 100 °C	12 psi (830 mbar) at 300 °C
APCI vaporiser gas (N_2)	20 psi (1380 mbar) at 350 °C	20 psi (1380 mbar) at 400 °C
APCI nebulising gas (N_2)	turned off	40 psi (2760 mbar)
**FT-ICR-MS parameters:**		
Ion Guide: Amplitude (V(b-p))	All materials: 240	240
Ion Guide: Frequency (MHz)	2.9	2.9
Ion Guide: Coil	2	2
Arbitrary Waveform: Amplitude (V(b-p))	180: copper resinate, matte dammar varnish, Prussian blue tempera paint, blackish brown real-life sample<br>150: Prussian blue oil paint	150
Arbitrary Waveform: DAC Rate (MHz)	8	8
Ion Signal: Acquisition Mode	Direct (Broadband)	Direct (Broadband)
Ion Signal: ADC Rate (MHz)	4	4
Ion Signal: Transient Length (ms)	524.288	524.288
Ion Signal: Number of Samples	2048 K	2048 K
Trapping Quadrupole: Width (ms)	3.8: copper resinate, matte dammar varnish, Prussian blue oil paint, Prussian blue tempera paint<br>3.6: blackish brown real-life sample	3.6

Table S2. Calibration standards used for external calibration in LA-APCI-FT-ICR-MS and APCI-FT-ICR-MS analysis. The used phosphazene cations are described in reference.[1]

Calculated *m/z*	Cation	
139.12297	$C_8H_{15}N_2^+$	1-butyl-3-methylimidazolium cation
257.18896	$C_{12}H_{26}N_4P^+$	Phosphazene cation
368.28149	$C_{14}H_{40}N_7P_2^+$	Phosphazene cation
552.28947	$C_{26}H_{45}N_7ClP_2^+$	Phosphazene cation
682.43526	$C_{26}H_{64}N_{13}P_4^+$	Phosphazene cation
916.57611	$C_{44}H_{82}N_{13}P_4^+$	Phosphazene cation

Table S3. Interpretation of mass spectra of aged copper resinate obtained with APCI-FT-ICR-MS from solution and LA-APCI-FT-ICR-MS from solid surface. Abbreviations: **CR** – copper resinate, **NA** – not assigned, **frag.** – fragment, **comp.** – compound.

**APCI-FT-ICR-MS (from solution)**		**LA-APCI-FT-ICR-MS (from solid surface)**		**Ion formula**		**Possible compound (M)**
**Measured *m/z***	**Δ*m/z* (ppm)**	**Measured *m/z***	**Δ*m/z* (ppm)**			
-	-	157.59807				NA
157.59916		-	-			NA
-	-	201.16402	1.19	$C_{15}H_{21}^+$		C15 frag. of CR
203.17935	-0.37	203.17940	-0.13	$C_{15}H_{23}^+$		C15 frag. of CR
-	-	205.19513	0.22	$C_{15}H_{25}^+$		C15 frag. of CR
-	-	210.47223				NA
219.17436	0.08	219.17442	0.34	$C_{15}H_{23}O^+$		C15 frag. of CR
-	-	221.19005	0.28	$C_{15}H_{25}O_+$		C15 frag. of CR
-	-	229.15883	0.61	$C_{16}H_{21}O^+$		C16 frag. of CR
-	-	233.15367	0.25	$C_{15}H_{21}O_2^+$		C15 frag. of CR
235.16908	-0.77	235.16934	0.32	$C_{15}H_{23}O_2^+$		C15 frag. of CR
-	-	237.18493	0.09	$C_{15}H_{25}O_2^+$		C15 frag. of CR
-	-	241.12220	-0.46	$C_{16}H_{17}O_2^+$		C16 frag. of CR
-	-	243.13803	0.30	$C_{16}H_{19}O_2^+$		C16 frag. of CR
-	-	245.15375	0.55	$C_{16}H_{21}O_2^+$		C16 fragment of CR
-	-	247.16942	0.65	$C_{16}H_{23}O_2^+$		C16 frag. of CR
-	-	249.14853	0.03	$C_{15}H_{21}O_3^+$		C15 frag. of CR
-	-	249.23307				NA
251.16424	0.29	-	-	$C_{15}H_{23}O_3^+$		C15 frag. of CR
-	-	251.17955	0.48	$C_{19}H_{23}^+$	$[M-H_2O-CO-2H+H]^+$	Didehydroabietic acid ($C_{20}H_{26}O_2$)
-	-	253.17991	0.36	$C_{15}H_{25}O_3^+$		C15 frag. of CR
253.19484	-0.96	253.19501	-0.29	$C_{19}H_{25}^+$	$[M-H_2O-CO+H]^+$	Didehydroabietic acid ($C_{20}H_{26}O_2$)
-	-	255.17413	-0.81	$C_{18}H_{23}O^+$		C18 frag. of CR
255.21022	-2.02	255.21071	-0.07	$C_{19}H_{27}^+$	$[M-H_2O-CO+H]^+$	Dehydroabietic acid ($C_{20}H_{28}O_2$)
-	-	257.22636	-0.07	$C_{19}H_{29}^+$	$[M-H_2O-CO+H]^+$	Abietic acid ($C_{20}H_{30}O_2$)
-	-	261.14861	0.33	$C_{16}H_{21}O_3^+$		C16 frag. of CR
-	-	265.15872	0.10	$C_{19}H_{21}O^+$		C19 frag. of CR
-	-	267.13784	-0.46	$C_{18}H_{19}O_2^+$		C18 frag. of CR
267.17415	-0.71	267.17430	-0.16	$C_{19}H_{23}O^+$	$[M-H_2O-CO-2H+H]^+$	7-oxodehydroabietic acid ($C_{20}H_{26}O_3$)
269.18975	-0.90	269.18998	-0.03	$C_{19}H_{25}O^+$	$[M-H_2O-CO+H]^+$	7-oxodehydroabietic acid ($C_{20}H_{26}O_3$)
271.20549	-0.56	271.20568	0.15	$C_{19}H_{27}O^+$	$[M-H_2O-CO+H]^+$	15-hydroxydehydroabietic acid ($C_{20}H_{28}O_3$)
273.18473	-0.65	273.18501	0.37	$C_{18}H_{25}O_2^+$		C18 frag. of CR
-	-	273.22141	0.42	$C_{19}H_{29}O^+$	$[M-H_2O-CO+O+H]^+$	Abietic acid ($C_{20}H_{30}O_2$)
275.16398	-0.68	275.16431	0.52	$C_{17}H_{23}O_3^+$		C17 frag. of CR
-	-	275.20038	-0.65	$C_{18}H_{27}O_2^+$		C18 frag. of CR
277.17977	-0.18	277.17981	-0.02	$C_{17}H_{25}O_3^+$		C17 frag. of CR
-	-	279.13772	-0.85	$C_{19}H_{19}O_2^+$		C19 frag. of CR
-	-	281.15345	-0.59	$C_{19}H_{21}O_2^+$		C19 frag. of CR
283.16938	0.42	283.16934	0.29	$C_{19}H_{23}O_2^+$	$[M-H_2O-CO-2H+H]^+$	15-hydroxy-7-oxodehydroabietic acid ($C_{20}H_{26}O_4$)
285.18473	-0.64	285.18488	-0.11	$C_{19}H_{25}O_2^+$	$[M-H_2O-CO+H]^+$	15-hydroxy-7-oxodehydroabietic acid ($C_{20}H_{26}O_4$)
-	-	285.22107	-0.77	$C_{20}H_{29}O^+$	$[M-H_2O+H]^+$	Abietic acid ($C_{20}H_{30}O_2$)
287.16417	0.00	287.16441	0.85	$C_{18}H_{23}O_3^+$		C18 frag. of CR
287.20040	-0.57	287.20050	-0.19	$C_{19}H_{27}O_2^+$	$[M-H_2O-CO+H]^+$	7,15-dihydroxydehydroabietic acid ($C_{20}H_{28}O_4$)
289.14332	-0.40	289.14376	1.09	$C_{17}H_{21}O_4^+$		C17 frag. of CR
289.17980	-0.07	289.17984	0.07	$C_{18}H_{25}O_3^+$		C18 frag. of CR
289.21596	-0.86	289.21647	0.89	$C_{19}H_{29}O_2^+$	$[M-H_2O-CO+2O+H]^+$	Abietic acid ($C_{20}H_{30}O_2$)
291.15898	-0.36	291.15904	-0.16	$C_{17}H_{23}O_4^+$		C17 frag. of CR
-	-	291.19523	-0.81	$C_{18}H_{27}O_3^+$		C18 frag. of CR

293.17449	-0.85	293.17481	0.24	$C_{17}H_{25}O_4^+$		C17 frag. of CR
297.18465	-0.87	297.18489	-0.06	$C_{20}H_{25}O_2^+$	$[M-H_2O+H]^+$	7-oxodehydroabietic acid ($C_{20}H_{26}O_3$)
299.16401	-0.54	299.16421	0.14	$C_{19}H_{23}O_3^+$	$[M-H_2O-CO-2H+O+H]^+$	15-hydroxy-7-oxodehydroabietic acid ($C_{20}H_{26}O_4$)
299.20030	-0.86	299.20044	-0.41	$C_{20}H_{27}O_2^+$	$[M+H]^+$	Didehydroabietic acid ($C_{20}H_{26}O_2$)
301.17961	-0.68	301.17982	0.00	$C_{19}H_{25}O_3^+$	$[M-H_2O-CO+O+H]^+$	15-hydroxy-7-oxodehydroabietic acid ($C_{20}H_{26}O_4$)
301.21596	-0.84	301.21616	-0.18	$C_{20}H_{29}O_2^+$	$[M+H]^+$	Dehydroabietic acid ($C_{20}H_{28}O_2$)
303.19529	-0.61	303.19553	0.20	$C_{19}H_{27}O_3^+$	$[M-H_2O-CO+O+H]^+$	7,15-dihydroxydehydroabietic acid ($C_{20}H_{28}O_4$)
-	-	303.23180	-0.20	$C_{20}H_{31}O_2^+$	$[M+H]^+$	Abietic acid ($C_{20}H_{30}O_2$)
305.17413	-2.00	305.17487	0.44	$C_{18}H_{25}O_4^+$		C18 frag. of CR
305.21075	-1.23	305.21117	0.17	$C_{19}H_{29}O_3^+$	$[M-H_2O-CO+3O+H]^+$	Abietic acid ($C_{20}H_{30}O_2$)
311.16398	-0.61	311.16408	-0.28	$C_{20}H_{23}O_3^+$	$[M-H_2O-2H+H]^+$	15-hydroxy-7-oxodehydroabietic acid ($C_{20}H_{26}O_4$)
-	-	311.54004				NA
313.17957	-0.81	313.17975	-0.21	$C_{20}H_{25}O_3^+$	$[M-H_2O+H]^+$	15-hydroxy-7-oxodehydroabietic acid ($C_{20}H_{26}O_4$)
315.15908	-0.04	315.15923	0.45	$C_{19}H_{23}O_4^+$	$[M-H_2O-CO-2H+2O+H]^+$	15-hydroxy-7oxodehydroabietic acid ($C_{20}H_{26}O_4$)
315.19526	-0.68	315.19532	-0.48	$C_{20}H_{27}O_3^+$	$[M+H]^+$	7-oxodehydroabietic acid ($C_{20}H_{26}O_3$)
317.17462	-0.37	317.17478	0.12	$C_{19}H_{25}O_4^+$	$[M-H_2O-CO+O2+H]^+$	15-hydroxy-7oxodehydroabietic acid ($C_{20}H_{26}O_4$)
317.20219		-	-			NA
317.21087	-0.80	317.21104	-0.27	$C_{20}H_{29}O_3^+$	$[M+O+H]^+$	Dehydroabietic acid ($C_{20}H_{28}O_2$)
319.19009	-0.94	319.19061	0.70	$C_{19}H_{27}O_4^+$	$[M-H_2O-CO+2O+H]^+$	7,15-dihydroxydehydroabietic acid ($C_{20}H_{28}O_4$)
319.22651	-0.81	319.22685	0.25	$C_{20}H_{31}O_3^+$	$[M+O+H]^+$	Abietic acid ($C_{20}H_{30}O_2$)
321.20586	-0.55	321.20620	0.51	$C_{19}H_{29}O_4^+$	$[M-H_2O-CO+4O+H]^+$	Abietic acid ($C_{20}H_{30}O_2$)
327.15877	-0.97	-	-	$C_{20}H_{23}O_4^+$	$[M-H_2O-2H+O+H]^+$	15-hydroxy-7-oxodehydroabietic acid ($C_{20}H_{26}O_4$)
329.17450	-0.74	329.17471	-0.10	$C_{20}H_{25}O_4^+$	$[M-2H+O+H]^+$	7-oxodehydroabietic acid ($C_{20}H_{26}O_3$)
329.21049	-1.92	-	-	$C_{21}H_{29}O_3^+$		Unknown
331.19006	-1.01	331.19026	-0.39	$C_{20}H_{27}O_4^+$	$[M+H]^+$	15-hydroxy-7-oxodehydroabietic acid ($C_{20}H_{26}O_4$)
333.16962	-0.08	333.16930	-1.04	$C_{19}H_{25}O_5^+$	$[M-H_2O-CO+3O+H]^+$	15-hydroxy-7oxodehydroabietic acid ($C_{20}H_{26}O_4$)
333.20569	-1.07	333.20587	-0.50	$C_{20}H_{29}O_4^+$	$[M+H]^+$	7,15-dihydroxydehyrdoabietic acid ($C_{20}H_{28}O_4$)
335.18511	-0.58	335.18526	-0.12	$C_{19}H_{27}O_5^+$	$[M-H_2O-CO+3O+H]^+$	7,15-dihydroxydehydroabietic acid ($C_{20}H_{28}O_4$)
335.22144	-0.76	335.22164	-0.16	$C_{20}H_{31}O_4^+$	$[M+2O+H]^+$	Abietic acid ($C_{20}H_{30}O_2$)
337.20068	-0.80	337.20089	-0.17	$C_{19}H_{29}O_5^+$	$[M-H_2O-CO+5O+H]^+$	Abietic acid ($C_{20}H_{30}O_2$)
343.22640	-1.09	-	-	$C_{22}H_{31}O_3^+$		Unknown
345.16947	-0.52	345.16968	0.10	$C_{20}H_{25}O_5^+$	$[M-2H+2O+H]^+$	7-oxodehydroabietic acid ($C_{20}H_{26}O_3$)
345.24236	-0.18	345.24225	-0.49	$C_{22}H_{33}O_3^+$		Unknown
347.18499	-0.89	347.18520	-0.28	$C_{20}H_{27}O_5^+$	$[M+O+H]^+$	15-hydroxy-7-oxodehydroabietic acid ($C_{20}H_{26}O_4$)
349.20065	-0.86	349.20091	-0.12	$C_{20}H_{29}O_5^+$	$[M+O+H]^+$	7,15-dihydroxydehyrdoabietic acid ($C_{20}H_{28}O_4$)
351.17988	-0.94	-	-	$C_{19}H_{27}O_6^+$	$[M-H_2O-CO+4O+H]^+$	7,15-dihydroxydehydroabietic acid ($C_{20}H_{28}O_4$)
351.21620	-1.15	351.21650	-0.27	$C_{20}H_{31}O_5^+$	$[M+3O+H]^+$	Abietic acid ($C_{20}H_{30}O_2$)
358.23738		-	-			NA
361.16421	-0.97	-	-	$C_{20}H_{25}O_6^+$	$[M-2H+2O+H]^+$	15-hydroxy-7-oxodehydroabietic acid ($C_{20}H_{26}O_4$)
363.17986	-0.97	363.17995	-0.72	$C_{20}H_{27}O_6^+$	$[M+2O+H]^+$	15-hydroxy-7-oxodehydroabietic acid ($C_{20}H_{26}O_4$)
365.19543	-1.20	365.19590	0.09	$C_{20}H_{29}O_6^+$	$[M+2O+H]^+$	7,15-dihydroxydehyrdoabietic acid ($C_{20}H_{28}O_4$)
367.21131	-0.57	367.21175	0.62	$C_{20}H_{31}O_6^+$	$[M+4O+H]^+$	Abietic acid ($C_{20}H_{30}O_2$)
373.23779	1.19	-	-	$C_{23}H_{33}O_4^+$		Unknown
375.21673	0.35	375.21709	1.29	$C_{22}H_{31}O_5^+$		Unknown
375.25269	-0.79	-	-	$C_{23}H_{35}O_4^+$		Unknown
381.19082	0.10	-	-	$C_{20}H_{29}O_7^+$	$[M+3O+H]^+$	7,15-dihydroxydehyrdoabietic acid ($C_{20}H_{28}O_4$)
391.24727	-1.61	-	-	$C_{23}H_{35}O_5^+$		Unknown
393.26399	1.12	-	-	$C_{23}H_{37}O_5^+$		Unknown
404.19836		-	-			NA

406.21410		-	-			NA
-	-	419.31527				NA
-	-	517.36691	-1.36	$C_{35}H_{49}O_3^+$		Combination of CR C20 comp. and C15 frag.
-	-	519.34636	-1.01	$C_{34}H_{47}O_4^+$		Combination of CR fragments (e.g., C19 and C15 frag.)
-	-	519.38265	-1.20	$C_{35}H_{51}O_3^+$		Combination of CR C20 comp. and C15 frag.
-	-	521.36379	2.39	$C_{34}H_{49}O_4^+$		Combination of CR fragments (e.g., C19 and C15 frag.)
-	-	531.34593	-1.81	$C_{35}H_{47}O_4^+$		Combination of CR C20 comp. and C15 frag.
533.36168	-1.61	533.36254	-0.01	$C_{35}H_{49}O_4^+$		Combination of CR C20 comp. and C15 frag.
-	-	535.34315	2.52	$C_{34}H_{47}O_5^+$		Combination of CR fragments (e.g., C19 and C15 frag.)
535.37897	1.46	535.37794	-0.47	$C_{35}H_{51}O_4^+$		Combination of CR C20 comp. and C15 frag.
-	-	537.35534				NA
-	-	537.39318	-1.22	$C_{35}H_{53}O_4^+$		Combination of CR C20 comp. and C15 frag.
-	-	539.37292	-0.33	$C_{34}H_{51}O_5^+$		Combination of CR fragments (e.g., C19 and C15 frag.)
-	-	547.34146	-0.61	$C_{35}H_{47}O_5^+$		Combination of CR C20 comp. and C15 frag.
549.35683	-1.12	549.35702	-0.79	$C_{35}H_{49}O_5^+$		Combination of CR C20 comp. and C15 frag.
-	-	551.33650	-0.41	$C_{34}H_{47}O_6^+$		Combination of CR fragments (e.g., C19 and C15 frag.)
551.37307	-0.06	551.37310	0.00	$C_{35}H_{51}O_5^+$		Combination of CR C20 comp. and C15 frag.
-	-	553.38895	0.35	$C_{35}H_{53}O_5^+$		Combination of CR C20 comp. and C15 frag.
-	-	561.35615	-2.31	$C_{36}H_{49}O_6^+$		Combination of CR C20 comp. and C16 frag.
-	-	565.35476				NA
-	-	565.36340				NA
-	-	567.37161				NA
-	-	567.37884				NA
-	-	569.39934	0.74	$C_{39}H_{53}O_3^+$	$[M-H_2O-CO+H]^+$	Combination of 7-oxodehydroabietic acid and dehydroabietic acid ($C_{40}H_{54}O_8$)
-	-	575.33710	0.66	$C_{36}H_{47}O_6^+$		Combination of CR C20 comp. and C16 frag.
-	-	575.37313	0.06	$C_{37}H_{51}O_5^+$		Combination of CR C20 comp. and C17 frag.
-	-	577.35248	0.18	$C_{36}H_{49}O_6^+$		Combination of CR C20 comp. and C16 frag.
-	-	581.35912				NA
-	-	583.37711	-1.84	$C_{39}H_{51}O_4^+$	$[M-H_2O-CO+H]^+$	Dimer of 7-oxodehydroabietic acid ($C_{40}H_{52}O_6$)
-	-	585.39350	-0.58	$C_{39}H_{53}O_4^+$	$[M-H_2O-CO+H]^+$	Combination of 7-oxodehydroabietic acid and 15-hydroxydehydroabietic acid ($C_{40}H_{54}O_6$)
-	-	587.33620	-0.88	$C_{37}H_{47}O_6^+$		Combination of CR C20 comp. and C17 frag.
-	-	587.37265	-0.76	$C_{38}H_{51}O_5^+$		Combination of CR C20 comp. and C18 frag.
-	-	587.40851	-1.66	$C_{39}H_{55}O_4^+$	$[M-H_2O-CO+H]^+$	Dimer of 15-hydroxydehydroabietic acid ($C_{40}H_{56}O_6$)
-	-	589.35243	0.10	$C_{37}H_{49}O_6^+$		Combination of CR C20 comp. and C17 frag.
-	-	591.36788	-0.24	$C_{37}H_{51}O_6^+$		Combination of CR C20 comp. and C17 frag.
-	-	593.38372	0.08	$C_{37}H_{53}O_6^+$		Combination of CR C20 comp. and C17 frag.
-	-	595.36543				NA
-	-	597.35599	-2.45	$C_{39}H_{49}O_5^+$	$[M-H_2O-CO-2H+H]^+$	Combination of 15-hydroxy-7-oxodehydroabietic acid and 7-oxodehydroabietic acid ($C_{40}H_{52}O_7$)
-	-	597.39370	-0.24	$C_{40}H_{53}O_4^+$	$[M+H]^+$	Dimer of didehydroabietic acid ($C_{40}H_{52}O_4$)
-	-	599.37179	-2.19	$C_{39}H_{51}O_5^+$	$[M-H_2O-CO+H]^+$	Combination of 15-hydroxy-7-oxodehydroabietic acid and 7-oxodehydroabietic acid ($C_{40}H_{52}O_7$)
-	-	599.40932	-0.28	$C_{40}H_{55}O_4^+$	$[M+H]^+$	Combination of didehydroabietic acid and dehydroabietic acid ($C_{40}H_{54}O_4$)
-	-	601.38861	-0.23	$C_{39}H_{53}O_5^+$	$[M-H_2O-CO+H]^+$	Combination of 15-hydroxydehydroabietic acid and 15-hydroxy-7-oxodehydroabietic acid ($C_{40}H_{54}O_7$)
-	-	601.42477	-0.62	$C_{40}H_{57}O_4^+$	$[M+H]^+$	Dimer of dehydroabietic acid ($C_{40}H_{56}O_4$)
-	-	603.33013	-2.48	$C_{37}H_{47}O_7^+$		Combination of CR C20 comp. and C17 frag.
-	-	603.36566				NA

-	-	603.40457	0.28	$C_{39}H_{55}O_5^+$	$[M-H_2O-CO+H]^+$	Combination of 15-hydroxydehydroabietic acid and 7,15-dihydroxydehydroabietic acid ($C_{40}H_{56}O_7$)
-	-	603.44003	-1.26	$C_{40}H_{59}O_4^+$	$[M+H]^+$	Combination of abietic acid and dehydroabietic acid ($C_{40}H_{58}O_4$)
-	-	605.34814	1.43	$C_{37}H_{49}O_7^+$		Combination of CR C20 comp. and C17 frag.
-	-	605.38440	1.21	$C_{38}H_{53}O_6^+$		Combination of CR C20 comp. and C18 frag.
-	-	607.36261	-0.52	$C_{37}H_{51}O_7^+$		Combination of CR C20 comp. and C17 frag.
-	-	609.34868				NA
-	-	609.35387				NA
-	-	609.37963	1.73	$C_{37}H_{53}O_7^+$		Combination of CR C20 comp. and C17 frag.
611.37286	-0.40	611.37236	-1.21	$C_{40}H_{51}O_5^+$	$[M-2H+H]^+$	Combination of 7-oxodehydroabietic acid and didehydroabietic acid ($C_{40}H_{52}O_5$)
-	-	613.35240	0.04	$C_{39}H_{49}O_6^+$	$[M-H_2O-CO-2H+O+H]^+$	Combination of 15-hydroxy-7-oxodehydroabietic acid and 7-oxodehydroabietic acid ($C_{40}H_{52}O_7$)
613.38814	-0.99	613.38833	-0.68	$C_{40}H_{53}O_5^+$	$[M+H]^+$	Combination of 7-oxodehydroabietic acid and didehydroabietic acid ($C_{40}H_{52}O_5$)
-	-	615.36685	-1.90	$C_{39}H_{51}O_6^+$	$[M-H_2O-CO+H]^+$	Dimer of 15-hydroxy-7-oxodehydroabietic acid ($C_{40}H_{52}O_8$)
615.40263	-2.88	615.40425	-0.24	$C_{40}H_{55}O_5^+$	$[M+H]^+$	Combination of dehydroabietic acid and 7-oxodehydroabietic acid ($C_{40}H_{54}O_5$)
-	-	617.38430	1.03	$C_{39}H_{53}O_6^+$	$[M-H_2O-CO+H]^+$	Combination of 7,15-dihydroxydehydroabietic acid and 15-hydroxy-7-oxodehydroabietic acid ($C_{40}H_{54}O_8$)
-	-	617.42032	0.44	$C_{40}H_{57}O_5^+$	$[M+H]^+$	Combination of dehydroabietic acid and 15-hydroxydehydroabietic acid ($C_{40}H_{56}O_5$)
-	-	619.40006	1.19	$C_{39}H_{55}O_6^+$	$[M-H_2O-CO+H]^+$	Dimer of 7,15-dihydroxydehydroabietic acid ($C_{40}H_{56}O_8$)
-	-	619.43577	0.11	$C_{40}H_{59}O_5^+$	$[M+H]^+$	Combination of abietic acid and 15-hydroxydehydroabietic acid ($C_{40}H_{58}O_5$)
-	-	621.41402	-1.54	$C_{39}H_{57}O_6^+$	$[M-H_2O-CO+2O+H]^+$	Combination of 7,15-dihydroxydehydroabietic acid and abietic acid ($C_{40}H_{58}O_6$)
-	-	623.35860	1.22	$C_{37}H_{51}O_8^+$		Combination of CR C20 comp. and C17 frag.
-	-	625.35134	-1.65	$C_{40}H_{49}O_6^+$	$[M-H_2O-2H+O+H]^+$	Dimer of 7-oxodehydroabietic acid ($C_{40}H_{52}O_6$)
-	-	625.37466	1.86	$C_{37}H_{53}O_8^+$		Combination of CR C20 comp. and C17 frag.
627.36732	-1.11	627.36802	-0.01	$C_{40}H_{51}O_6^+$	$[M-2H+H]^+$	Dimer of 7-oxodehydroabietic acid ($C_{40}H_{52}O_6$)
-	-	629.34592	-2.17	$C_{39}H_{49}O_7^+$	$[M-H_2O-CO-2H+O+H]^+$	Dimer of 15-hydroxy-7-oxodehydroabietic acid ($C_{40}H_{52}O_8$)
629.38258	-1.74	629.38288	-1.26	$C_{40}H_{53}O_6^+$	$[M+H]^+$	Dimer of 7-oxodehydroabietic acid ($C_{40}H_{52}O_6$)
-	-	631.36239	-0.85	$C_{39}H_{51}O_7^+$	$[M-H_2O-CO+O+H]^+$	Dimer of 15-hydroxy-7-oxodehydroabietic acid ($C_{40}H_{52}O_8$)
631.39865	-1.06	631.39907	-0.39	$C_{40}H_{55}O_6^+$	$[M+H]^+$	Combination of 7-oxodehydroabietic acid and 15-hydroxydehydroabietic acid ($C_{40}H_{54}O_6$)
-	-	633.37822	-0.57	$C_{39}H_{53}O_7^+$	$[M-H_2O-CO+O+H]^+$	Combination of 7,15-dihydroxydehydroabietic acid and 15-hydroxy-7-oxodehydroabietic acid ($C_{40}H_{54}O_8$)
633.41452	-0.72	633.41540	0.67	$C_{40}H_{57}O_6^+$	$[M+H]^+$	Dimer of 15-hydroxydehydroabietic acid ($C_{40}H_{56}O_6$)
-	-	635.39506	1.31	$C_{39}H_{55}O_7^+$	$[M-H_2O-CO+O+H]^+$	Dimer of 7,15-dihydroxydehydroabietic acid ($C_{40}H_{56}O_8$)
-	-	635.43130	1.07	$C_{40}H_{59}O_6^+$	$[M+H]^+$	Combination of abietic acid and 15-hydroxydehydroabietic acid ($C_{40}H_{58}O_6$)
-	-	643.36384	1.41	$C_{40}H_{51}O_7^+$	$[M-2H+H]^+$	Combination of 7-oxodehydroabietic acid and 15-hydroxy-7-oxodehydroabietic acid ($C_{40}H_{52}O_7$)
645.37839	-0.30	645.37866	0.13	$C_{40}H_{53}O_7^+$	$[M+H]^+$	Combination of 7-oxodehydroabietic acid and 15-hydroxy-7-oxodehydroabietic acid ($C_{40}H_{52}O_7$)
-	-	647.35715	-1.07	$C_{39}H_{51}O_8^+$	$[M-H_2O-CO+2O+H]^+$	Dimer of 15-hydroxy-7-oxodehydroabietic acid ($C_{40}H_{52}O_8$)

647.39444	0.32	647.39444	0.32	$C_{40}H_{55}O_7^+$	$[M+H]^+$	Combination of 15-hydroxydehydroabietic acid and 15-hydroxy-7-oxodehydroabietic acid ($C_{40}H_{54}O_7$)
-	-	649.37437	1.34	$C_{39}H_{53}O_8^+$	$[M-H_2O-CO+2O+H]^+$	Combination of 7,15-dihydroxydehydroabietic acid and 15-hydroxy-7-oxodehydroabietic acid ($C_{40}H_{54}O_8$)
-	-	649.41030	0.65	$C_{40}H_{57}O_7^+$	$[M+H]^+$	Combination of 15-hydroxydehydroabietic acid and 7,15-dihydroxydehydroabietic acid ($C_{40}H_{56}O_7$)
-	-	651.38923	0.12	$C_{39}H_{55}O_8^+$	$[M-H_2O-CO+2O+H]^+$	Dimer of 7,15-dihydroxydehydroabietic acid ($C_{40}H_{56}O_8$)
-	-	651.42740	2.87	$C_{40}H_{59}O_7^+$	$[M+O+H]^+$	Combination of abietic acid and 7,15-dihydroxydehydroabietic acid ($C_{40}H_{58}O_6$)
-	-	661.37327	-0.34	$C_{40}H_{53}O_8^+$	$[M+H]^+$	Dimer of 15-hydroxy-7-oxodehydroabietic acid ($C_{40}H_{52}O_8$)
663.38820	-1.44	663.38901	-0.21	$C_{40}H_{55}O_8^+$	$[M+H]^+$	Combination of 15-hydroxy-7-oxodehydroabietic acid and 7,15-dihydroxydehydroabietic acid ($C_{40}H_{54}O_8$)
-	-	665.40452	-0.43	$C_{40}H_{57}O_8^+$	$[M+H]^+$	Dimer of 7,15-dihydroxydehydroabietic acid ($C_{40}H_{56}O_8$)
-	-	667.42017	-0.42	$C_{40}H_{59}O_8^+$	$[M+2O+H]^+$	Combination of abietic acid and 7,15-dihydroxydehydroabietic acid ($C_{40}H_{58}O_6$)
-	-	677.37048				NA
-	-	679.38534	1.89	$C_{40}H_{55}O_9^+$	$[M+O+H]^+$	Combination of 15-hydroxy-7-oxodehydroabietic acid and 7,15-dihydroxydehydroabietic acid ($C_{40}H_{54}O_8$)
-	-	681.40002	0.45	$C_{40}H_{57}O_9^+$	$[M+O+H]^+$	Dimer of 7,15-dihydroxydehydroabietic acid ($C_{40}H_{56}O_8$)
-	-	683.41496	-0.58	$C_{40}H_{59}O_9^+$	$[M+3O+H]^+$	Combination of abietic acid and 7,15-dihydroxydehydroabietic acid ($C_{40}H_{58}O_8$)

Table S4. Interpretation of the mass spectrum of Prussian blue linseed oil paint obtained with LA-APCI-FT-ICR-MS.

Abbreviations of fatty acids, dicarboxylic acids and other degradation products of triacylglycerols (**TAGs**), diacylglycerols (**DAGs**): **Ln** = linolenic acid ($C_{18}H_{30}O_2$); **L** = linoleic acid ($C_{18}H_{32}O_2$); **O** = oleic acid ($C_{18}H_{34}O_2$); **S** = stearic acid ($C_{18}H_{36}O_2$); **P** = palmitic acid ($C_{16}H_{32}O_2$); **Po** = palmitoleic acid ($C_{16}H_{30}O_2$); **Ar** = arachidic acid ($C_{20}H_{40}O_2$); **Eic** = eicosenoic acid ($C_{20}H_{38}O_2$); **Myr** =myristic acid ($C_{14}H_{28}O_2$); **C12:0** = lauric acid ($C_{12}H_{24}O_2$); **DDDA** = dodecanedioic acid ($C_{12}H_{22}O_4$); **C11:1** = 10-undecenoic acid ($C_{11}H_{20}O_2$); **UDDA** = undecanedioic acid ($C_{11}H_{20}O_4$); **Se** = sebacic acid ($C_{10}H_{18}O_4$); **C10:2** = decadienoic acid ($C_{10}H_{16}O_2$); **C10:0** = capric acid ($C_{10}H_{20}O_2$); **Az** = azelaic acid ($C_9H_{16}O_4$); **9-ONA** = 9-oxononanoic acid ($C_9H_{16}O_3$); **C9:1** = 2-nonenoic acid ($C_9H_{16}O_2$); **C9:0** = nonanoic acid ($C_9H_{18}O_2$); **Su** = suberic acid ($C_8H_{14}O_4$); **8-OOA** = 8-oxooctanoic acid ($C_8H_{14}O_3$); **C8:1** = octenoic acid ($C_8H_{14}O_2$); **C8:0** = octanoic acid ($C_8H_{16}O_2$); **Pi** = pimelic acid ($C_7H_{12}O_4$); **C7:1** = heptenoic acid ($C_7H_{12}O_2$); **C7:0** = heptanoic acid ($C_7H_{14}O_2$); **C6:1** = hexenoic acid ($C_6H_{10}O_2$); **C6:0** = hexanoic acid ($C_6H_{12}O_2$); **GA** = glutaric acid ($C_5H_8O_4$); **Suc** = succinic acid ($C_4H_6O_4$); **MA** = Malonic acid ($C_3H_4O_4$); **Oxa** = Oxalic acid ($C_2H_2O_4$). The abbreviation of monoacylglycerol is **MAG**. All degradation products have been found in different studies.[2–7]

Measured *m/z*	Δ*m/z* (ppm)	Ion formula		Possible compound (M)
257.24757	0.23	$C_{16}H_{33}O_2^+$	$[M+H]^+$	Palmitic acid
265.25283	0.90	$C_{18}H_{33}O^+$	$[M-H_2O+H]^+$	Oleic acid
285.27885	0.14	$C_{18}H_{37}O_2^+$	$[M+H]^+$	Stearic acid
313.27373	0.03	$C_{19}H_{37}O_3^+$	$[M-H_2O+H]^+$	**MAG:** Glyceryl monopalmitate
339.21652	-0.24	$C_{19}H_{31}O_5^+$	$[M+H]^+$/ $[M-H_2O+H]^+$	**DAG:** C10:2C6:1/ 9-ONAC7:1
339.28934	-0.09	$C_{21}H_{39}O_3^+$	$[M-H_2O+H]^+$	**MAG:** Glyceryl monooleate
341.19602	0.44	$C_{18}H_{29}O_6^+$	$[M-H_2O+H]^+$	**DAG:** PiC8:1/ SuC7:1/ AzC6:1
341.23202	-0.67	$C_{19}H_{33}O_5^+$	$[M+H]^+$/ $[M+H]^+$/ $[M-H_2O+H]^+$	**DAG:** C10:2C6:0/ C9:1C7:1/ 9-ONAC7:0
341.30493	-0.26	$C_{21}H_{41}O_3^+$	$[M-H_2O+H]^+$	**MAG:** Glyceryl monostearate
353.23231	0.17	$C_{20}H_{33}O_5^+$	$[M+H]^+$/ $[M-H_2O+H]^+$/ $[M-2H_2O+H]^+$	**DAG:** C10:2C7:1/ 8-OOAC9:1/ PiC10:0
353.26839	-0.71	$C_{21}H_{37}O_4^+$	$[M+H]^+$	**MAG:** Glyceryl monolinolenate
355.21149	-0.08	$C_{19}H_{31}O_6^+$	$[M-H2O+H]^+$	**DAG:** PiC9:1/ SeC6:1/ AzC7:1
355.24780	-0.28	$C_{20}H_{35}O_5^+$	$[M+H]^+$	**DAG:** C10:2C7:0/ C11:1C6:1
355.28400	-0.82	$C_{21}H_{39}O_4^+$	$[M+H]^+$	**MAG:** Glyceryl monolinoleate
365.23232	0.19	$C_{21}H_{33}O_5^+$	$[M-H_2O+H]^+$/ $[M-2H_2O+H]^+$/ $[M-2H_2O+H]^+$	**DAG:** 8-OOAC10:2/ AzC9:1/ 2x9-ONA
367.21158	0.16	$C_{20}H_{31}O_6^+$	$[M-H_2O+H]^+$/ $[M-2H_2O+H]^+$/ $[M-2H_2O+H]^+$	**DAG:** PiC10:2/ Su9-ONA/ Az8-OOA
367.24784	-0.16	$C_{21}H_{35}O_5^+$	$[M+H]^+$ /$[M-H_2O+H]^+$/ $[M-2H_2O+H]^+$	**DAG:** C10:2C8:1/ 9-ONAC9:1/ SuC10:0
369.22723	0.16	$C_{20}H_{33}O_6^+$	$[M-H_2O+H]^+$	**DAG:** SuC9:1/ 8-OOA9-ONA
371.24275	-0.19	$C_{20}H_{35}O_6^+$	$[M+H]^+$/ $[M-H_2O+H]^+$/ $[M-H_2O+H]^+$/ $[M-H_2O+H]^+$	**DAG:** 8-OOAC9:1/ PiC10:0/ AzC8:0/ SuC9:0
379.24758	-0.84	$C_{22}H_{35}O_5^+$	$[M-H_2O+H]^+$/ $[M-2H_2O+H]^+$	**DAG:** 9-ONAC10:2/ SeC9:1
381.22708	-0.24	$C_{21}H_{33}O_6^+$	$[M-H_2O+H]^+$/ $[M-2H_2O+H]^+$/ $[M-2H_2O+H]^+$	**DAG:** SuC10:2/ Se8-OOA/ Az9-ONA
381.26371	0.42	$C_{22}H_{37}O_5^+$	$[M+H]^+$/ $[M-2H_2O+H]^+$	**DAG:** C10:2C9:1/AzC10:0
383.24268	-0.37	$C_{21}H_{35}O_6^+$	$[M+H]^+$/ $[M-H_2O+H]^+$/ $[M-H_2O+H]^+$	**DAG:** 8-OOAC10:2/ AzC9:1/ 2x9-ONA
385.22152	-1.45	$C_{20}H_{33}O_7^+$	$[M+H]^+$ /$[M-H_2O+H]^+$/ $[M-H_2O+H]^+$	**DAG:** PiC10:2/ Su9-ONA/ Az8-OOA
393.26380	0.64	$C_{23}H_{37}O_5^+$	$[M+H]^+$/ $[M-2H_2O+H]^+$/ $[M-2H_2O+H]^+$	**DAG:** 2xC10:2/ PoSuc/ AzC11:1
393.29985	-0.23	$C_{24}H_{41}O_4^+$	$[M-2H_2O+H]^+$	**DAG:** 9-ONAC12:0
395.24271	-0.28	$C_{22}H_{35}O_6^+$	$[M-H_2O+H]^+$/$[M-2H_2O+H]^+$	**DAG:** AzC10:2/ Se9-ONA
395.31565	0.15	$C_{24}H_{43}O_4^+$	$[M-H_2O+H]^+$	**DAG:** C9:1C12:0/ MyrC7:1
397.22193	-0.38	$C_{21}H_{33}O_7^+$	$[M-2H_2O+H]^+$/ $[M-2H_2O+H]^+$	**DAG:** AzAz/ SeSu
397.25845	-0.05	$C_{22}H_{37}O_6^+$	$[M+H]^+$/ $[M-H_2O+H]^+$	**DAG:** 9-ONAC10:2/ SeC9:1
399.23789	0.40	$C_{21}H_{35}O_7^+$	$[M+H]^+$/ $[M-H_2O+H]^+$/ $[M-H_2O+H]^+$	**DAG:** SuC10:2/ Se8-OOA/ Az9-ONA
407.24274	-0.20	$C_{23}H_{35}O_6^+$	$[M-3H_2O+H]^+$/ $[M-H_2O+H]^+$	**DAG:** SeSe/ LnOxa
407.27904	-0.39	$C_{24}H_{39}O_5^+$	$[M-2H_2O+H]^+$	**DAG:** SeC11:1
409.25824	-0.56	$C_{23}H_{37}O_6^+$	$[M-H_2O+H]^+$	**DAG:** SeC10:2/ LOxa
409.29470	-0.37	$C_{24}H_{41}O_5^+$	$[M+H]^+$/ $[M-2H_2O+H]^+$/ $[M-2H_2O+H]^+$	**DAG:** C11:1C10:2/ AzC12:0/ MyrPi
411.23765	-0.19	$C_{22}H_{35}O_7^+$	$[M-2H_2O+H]^+$	**DAG:** AzSe
411.27398	-0.34	$C_{23}H_{39}O_6^+$	$[M-H_2O+H]^+$	**DAG:** AzC11:1
413.25333	-0.12	$C_{22}H_{37}O_7^+$	$[M+H]^+$/$[M-H_2O+H]^+$	**DAG:** AzC10:2/ Se9-ONA
421.25849	0.05	$C_{24}H_{37}O_6^+$	$[M-H_2O+H]^+$	**DAG:** LnMA
421.29400	-2.02	$C_{25}H_{41}O_5^+$	$[M-2H_2O+H]^+$	**DAG:** EicOxa/ OSuc
423.23766	-0.17	$C_{23}H_{35}O_7^+$	$[M+O-3H_2O+H]^+$	**DAG:** SeSe
423.27393	-0.45	$C_{24}H_{39}O_6^+$	$[M-H_2O+H]^+$/ $[M-2H_2O+H]^+$/ $[M-2H_2O+H]^+$	**DAG:** UDDAC10:2/ DDDA9-ONA
423.34623	-1.56	$C_{26}H_{47}O_4^+$	$[M-H_2O+H]^+$	**DAG:** PC7:1/ MyrC9:1
425.25405	1.58	$C_{23}H_{37}O_7^+$	$[M-2H_2O+H]^+$	**DAG:** SeSe
435.23702	-1.63	$C_{24}H_{35}O_7^+$	$[M+2O-2H_2O+H]^+$	**DAG:** LnMA
435.27468	1.29	$C_{25}H_{39}O_6^+$	$[M-H_2O+H]^+$	**DAG:** LnSuc

437.25306	-0.73	$C_{24}H_{37}O_7^+$	$[M+O-H_2O+H]^+$	**DAG:** LnMA
437.29027	1.14	$C_{25}H_{41}O_6^+$	$[M-H_2O+H]^+$	**DAG:** LSuc/ DDDAC10:2
437.36245	-0.21	$C_{27}H_{49}O_4^+$	$[M-H_2O+H]^+$	**DAG:** OC6:0/ SC6:1/ PC8:1/ PoC8:0
439.26964	1.39	$C_{24}H_{39}O_7^+$	$[M+H]^+$	**DAG:** LnMA/ AzDDDA
439.34144	-0.82	$C_{26}H_{47}O_5^+$	$[M-H_2O+H]^+$	**DAG:** Myr9-ONA
439.37806	-0.30	$C_{27}H_{51}O_4^+$	$[M-H_2O+H]^+$	**DAG:** SC6:0/ PC8:0/ MyrC10:0
449.28997	0.45	$C_{26}H_{41}O_6^+$	$[M-H_2O+H]^+$	**DAG:** LnGA
451.26961	1.29	$C_{25}H_{39}O_7^+$	$[M+O-H_2O+H]^+$	**DAG:** LnSuc
451.34180	0.00	$C_{27}H_{47}O_5^+$	$[M+H]^+$/ $[M+H]^+$/ $[M-H_2O+H]^+$	**DAG:** LnC6:0/ LC6:1/ Po8-OOA
451.37798	-0.47	$C_{28}H_{51}O_4^+$	$[M-H_2O+H]^+$	**DAG:** PC9:1/ PoC9:0/ OC7:0/ SC7:1
453.35738	-0.15	$C_{27}H_{49}O_5^+$	$[M+H]^+$/ $[M+H]^+$/ $[M+H]^+$/ $[M-H_2O+H]^+$	**DAG:** LC6:0/ OC6:1/ PoC8:1/ P8-OOA
455.26402	0.18	$C_{24}H_{39}O_8^+$	$[M+O+H]^+$	**DAG:** LnMA
463.26892	-0.24	$C_{26}H_{39}O_7^+$	$[M+2O-2H_2O+H]^+$	**DAG:** LnGA
463.37790	-0.63	$C_{29}H_{51}O_4^+$	$[M-H_2O+H]^+$/ $[M-H_2O+H]^+$/ $[M-2H_2O+H]^+$	**DAG:** LC8:0/ OC8:1/ S8-OOA
465.28396	-1.55	$C_{26}H_{41}O_7^+$	$[M+O-H_2O+H]^+$	**DAG:** LnGA
465.35688	-1.22	$C_{28}H_{49}O_5^+$	$[M+H]^+$/ $[M-H_2O+H]^+$/ $[M-2H_2O+H]^+$/ $[M-2H_2O+H]^+$	**DAG:** LnC7:0/ Po9-ONA/ PAz/ SPi
465.39400	0.34	$C_{29}H_{53}O_4^+$	$[M-H_2O+H]^+$	**DAG:** PoC10:0/ OC8:0/ SC8:1
467.37269	-0.88	$C_{28}H_{51}O_5^+$	$[M+H]^+$/ $[M-H_2O+H]^+$	**DAG:** PoC9:1/ P9-ONA
467.40903	-0.98	$C_{29}H_{55}O_4^+$	$[M-H_2O+H]^+$	**DAG:** PC10:0/ SC8:0
477.35664	-1.70	$C_{29}H_{49}O_5^+$	$[M+H]^+$/ $[M-H_2O+H]^+$/ $[M-2H_2O+H]^+$/ $[M-2H_2O+H]^+$	**DAG:** LnC8:1/ L8-OOA/ OSu/ PoSe
479.37308	-0.04	$C_{29}H_{51}O_5^+$	$[M+H]^+$/ $[M+H]^+$/ $[M-H_2O+H]^+$/ $[M-2H_2O+H]^+$/ $[M-2H_2O+H]^+$	**DAG:** LnC8:0/ LC8:1/ O8-OOA/ SSu/ PSe
481.35204	-0.69	$C_{28}H_{49}O_6^+$	$[M-H_2O+H]^+$	**DAG:** PoAz/ OPi
481.38841	-0.71	$C_{29}H_{53}O_5^+$	$[M+H]^+$/ $[M+H]^+$/ $[M-H_2O+H]^+$	**DAG:** LC8:0/ OC8:1/ S8-OOA
483.36786	-0.33	$C_{28}H_{51}O_6^+$	$[M+H]^+$/ $[M-H_2O+H]^+$/ $[M-H_2O+H]^+$	**DAG:** Po9-ONA/ PAz/ SPi
491.37279	-0.63	$C_{30}H_{51}O_5^+$	$[M+H]^+$/ $[M-H_2O+H]^+$/ $[M-2H_2O+H]^+$	**DAG:** LnC9:1/ L9-ONA /OAz
493.35281	0.89	$C_{29}H_{49}O_6^+$	$[M+H]^+$/ $[M-H_2O+H]^+$	**DAG:** Ln8-OOA/ LSu
493.38837	-0.77	$C_{30}H_{53}O_5^+$	$[M+H]^+$/ $[M+H]^+$/ $[M-H_2O+H]^+$/ $[M-2H_2O+H]^+$	**DAG:** LC9:1/ LnC9:0/ O9-ONA/ SAz
495.36768	-0.69	$C_{29}H_{51}O_6^+$	$[M+H]^+$/ $[M-H_2O+H]^+$/ $[M-H_2O+H]^+$	**DAG:** L8-OOA/ OSu/ PoSe
495.40380	-1.21	$C_{30}H_{55}O_5^+$	$[M+H]^+$/ $[M+H]^+$/ $[M-H_2O+H]^+$	**DAG:** OC9:1/ LC9:0/ S9-ONA
497.38305	-1.25	$C_{29}H_{53}O_6^+$	$[M+H]^+$/ $[M-H_2O+H]^+$/ $[M-H_2O+H]^+$	**DAG:** O8-OOA/ SSu/ PSe
505.35261	0.47	$C_{30}H_{49}O_6^+$	$[M-H_2O+H]^+$	**DAG:** LnAz
505.38870	-0.10	$C_{31}H_{53}O_5^+$	$[M+H]^+$/ $[M-2H_2O+H]^+$/ $[M-2H_2O+H]^+$	**DAG:** LC10:2/ OSe/ EicSu
507.33061	-2.01	$C_{29}H_{47}O_7^+$	$[M+O-H_2O+H]^+$	**DAG:** LnSu
507.36785	-0.34	$C_{30}H_{51}O_6^+$	$[M+H]^+$/$[M-H_2O+H]^+$	**DAG:** Ln9-ONA/ LAz
507.40402	-0.75	$C_{31}H_{55}O_5^+$	$[M+H]^+$/ $[M+H]^+$/ $[M-2H_2O+H]^+$	**DAG:** LnC10:0/ OC10:2/ SSe
509.38315	-1.02	$C_{30}H_{53}O_6^+$	$[M+H]^+$/ $[M-H_2O+H]^+$	**DAG:** L9-ONA/ OAz
509.41963	-0.82	$C_{31}H_{57}O_5^+$	$[M+H]^+$/$[M+H]^+$/ $[M+H]^+$	**DAG:** LC10:0/ SC10:2/ EicC8:1
511.39896	-0.70	$C_{30}H_{55}O_6^+$	[M+H]+/ $[M-H_2O+H]^+$	**DAG:** O9-ONA/ SAz
517.31677	1.53	$C_{30}H_{45}O_7^+$	$[M+3O-3H_2O+H]^+$	**DAG:** LnAz
517.38791	-1.62	$C_{32}H_{53}O_5^+$	$[M-2H_2O+H]^+$	**DAG:** LUDDA
519.33169	0.12	$C_{30}H_{47}O_7^+$	$[M+2O-2H_2O+H]^+$	**DAG:** LnAz
519.36783	-0.37	$C_{31}H_{51}O_6^+$	$[M-H_2O+H]^+$	**DAG:** LnSe
519.40454	0.27	$C_{32}H_{55}O_5^+$	$[M-2H_2O+H]^+$	**DAG:** EicAz
521.34721	-0.13	$C_{30}H_{49}O_7^+$	$[M+O-H_2O+H]^+$	**DAG:** LnAz
521.38353	-0.27	$C_{31}H_{53}O_6^+$	$[M-H_2O+H]^+$	**DAG:** LSeb
521.42001	-0.08	$C_{32}H_{57}O_5^+$	$[M-2H_2O+H]^{+/}$ $[M-H_2O+H]^+$	**DAG:** ArAz/ Eic9-ONA
523.36213	-1.53	$C_{30}H_{51}O_7^+$	$[M+H]^+$	**DAG:** LnAz
523.39896	-0.69	$C_{31}H_{55}O_6^+$	$[M-H_2O+H]^+$	**DAG:** OSe/ EicSu
523.43544	-0.50	$C_{32}H_{59}O_5^+$	$[M+H]^+$/ $[M-H_2O+H]^+$	**DAG:** EicC9:1/ Ar9-ONA
533.31076	-0.24	$C_{30}H_{45}O_8^+$	$[M+4O-3H_2O+H]^+$	**DAG:** LnAz
533.38347	-0.37	$C_{32}H_{53}O_6^+$	$[M-H_2O+H]^+$	**DAG:** LnUDDA
533.42068	1.18	$C_{33}H_{57}O_5^+$	$[M+O-H_2O+H]^+$	**DAG:** LnC12:0
535.39921	-0.21	$C_{32}H_{55}O_6^+$	$[M-H_2O+H]^+$	**DAG:** L UDDA
537.37731	-2.36	$C_{31}H_{53}O_7^+$	$[M+H]^+$	**DAG:** LnSe
537.41429	-1.27	$C_{32}H_{57}O_6^+$	$[M-H_2O+H]$+	**DAG:** EicAz
539.39378	-0.83	$C_{31}H_{55}O_7^+$	$[M+H]^+$/ $[M+H]^+$	**DAG:** LSe
547.39934	0.04	$C_{33}H_{55}O_6^+$	$[M-H_2O+H]^+$	**DAG:** LnDDDA
549.41462	-0.64	$C_{33}H_{57}O_6^+$	$[M-H_2O+H]^+$/ $[M+O-2H_2O+H]^+$	**DAG:** L DDDA/ EicSe
551.50305	-0.62	$C_{35}H_{67}O_4^+$	$[M-H_2O+H]^+$	**DAG:** PP/ SMyr
553.33734	0.42	$C_{30}H_{49}O_9^+$	$[M+3O-H_2O+H]^+$	**DAG:** LnAz
561.41435	-1.10	$C_{34}H_{57}O_6^+$	$[M+2O-3H_2O+H]^+$	**DAG:** EicUDDA
563.39407	-0.28	$C_{33}H_{55}O_7^+$	$[M+O-H_2O+H]^+$	**DAG:** LnDDDA

563.43152	1.60	$C_{34}H_{59}O_6^+$	$[M+O-2H_2O+H]^+$	**DAG:** EicUDDA
565.40973	-0.27	$C_{33}H_{57}O_7^+$	$[M+O-H_2O+H]^+$	**DAG:** LDDDA
575.50301	-0.66	$C_{37}H_{67}O_4^+$	$[M-H_2O+H]^+$	**DAG:** LP/ OPo
577.51878	-0.45	$C_{37}H_{69}O_4^+$	$[M-H_2O+H]^+$	**DAG:** OP/ SPo
579.53435	-0.59	$C_{37}H_{71}O_4^+$	$[M-H_2O+H]^+$	**DAG:** SP
589.48226	-0.66	$C_{37}H_{65}O_5^+$	$[M+H]^+$	**DAG:** LnPo
591.49790	-0.68	$C_{37}H_{67}O_5^+$	$[M+H]^+$	**DAG:** LnP/ LPo
593.51359	-0.61	$C_{37}H_{69}O_5^+$	$[M+H]^+$	**DAG:** LP/ OPo
601.51845	-0.98	$C_{39}H_{69}O_4^+$	$[M-H_2O+H]^+$	**DAG:** LnS/ LO
603.46315	2.04	$C_{37}H_{63}O_6^+$	**DAG:** $[M-H_2O+2O+H]^+$<br>**TAG:** $[M-2H_2O+H]^+$	**DAG:** LnPo<br>**TAG:** PoSeC8:0/ PoAzC9:0
603.53473	0.07	$C_{39}H_{71}O_4^+$	$[M-H_2O+H]^+$	**DAG:** LS/ OO/ PoEic
605.47725	-0.53	$C_{37}H_{65}O_6^+$	**DAG:** $[M+O+H]^+$<br>**TAG:** $[M-2H_2O+H]^+$	**DAG:** LnPo<br>**TAG:** PSeC8:0/ PAzC9:0
605.55044	0.17	$C_{39}H_{73}O_4^+$	$[M-H_2O+H]^+$	**DAG:** SO/ PoAr/ PEic
607.56619	0.33	$C_{39}H_{75}O_4^+$	$[M-H_2O+H]^+$	**DAG:** SS/ PAr
615.49895	1.06	$C_{39}H_{67}O_5^+$	$[M+H]^+$	**DAG:** LLn
617.44081	-0.60	$C_{37}H_{61}O_7^+$	$[M+H]^+$/ $[M-H_2O+H]^+$	**TAG:** Ln8-OOAC8:1/ LnSuC8:0
617.51337	-0.94	$C_{39}H_{69}O_5^+$	$[M+H]^+$	**DAG:** LnO/ LL
619.45639	-0.71	$C_{37}H_{63}O_7^+$	**DAG:** $[M-H_2O+3O+H]^+$<br>**TAG:** $[M-H_2O+H]^+$	**DAG:** LnPo<br>**TAG:** PoAzC9:1/ LSuC8:0/ PSuC10:2
619.52956	-0.06	$C_{39}H_{71}O_5^+$	$[M+H]^+$	**DAG:** LnS/ LO
621.54613	1.42	$C_{39}H_{73}O_5^+$	$[M+H]^+$	**DAG:** LS/ OO
629.47701	-0.89	$C_{39}H_{65}O_6^+$	**DAG:** $[M+O+H]^+$<br>**TAG:** $[M+H]^+$/ $[M-2H_2O+H]^+$/ $M-2H_2O+H]^+$	**DAG:** LnLn<br>**TAG:** Po2xC10:2/ LSeC8:0/ LAzC9:0
631.49427	1.66	$C_{39}H_{67}O_6^+$	**DAG:** $[M+O+H]^+$<br>**TAG:** $[M+H]^+$/ $[M-2H_2O+H]^+$/ $[M-2H_2O+H]^+$	**DAG:** LLn<br>**TAG:** P2xC10:2/ OSeC8:0/ OAzC9:0
633.47237	-0.17	$C_{38}H_{65}O_7^+$	$[M-H_2O+H]^+$	**TAG:** LAzC8:0/ LSuC9:0/ PAzC10:2
633.50816	-1.12	$C_{39}H_{69}O_6^+$	**DAG:** $[M+O+H]^+$<br>**TAG:** $[M+H]^+$/ $[M-2H_2O+H]^+$/ $[M-2H_2O+H]^+$	**DAG:** LnO/ LL<br>**TAG:** PoC10:0C10:2/ SSeC8:0/ SAzC9:0
635.52500	0.76	$C_{39}H_{71}O_6^+$	**DAG:** $[M+O+H]^+$<br>**TAG:** $[M+H]^+$	**DAG:** LnS/ LO<br>**TAG:** PC10:0C10:2
637.46692	-0.75	$C_{37}H_{65}O_8^+$	**DAG:** $[M+3O+H]^+$<br>**TAG:** $[M+H]^+$	**DAG:** LnPo<br>**TAG:** PoAzC9:1/ LSuC8:0/ PSuC10:2
645.47271	0.36	$C_{39}H_{65}O_7^+$	**DAG:** $[M+2O+H]^+$<br>**TAG:** $[M+H]^+$/ $[M-H_2O+H]^+$/ $[M-H2O+H]^+$/<br>$[M-H_2O+H]^+$	**DAG:** LnLn<br>**TAG:** Ln9-ONAC9:1/ LAzC9:1/ LnSeC8:0/<br>LnAzC9:0
647.48879	1.02	$C_{39}H_{67}O_7^+$	**DAG:** $[M+2O+H]^+$<br>**TAG:** $[M+H]^+$/ $[M-H_2O+H]^+$/ $[M-H2O+H]^+$/<br>$[M-H_2O+H]^+$	**DAG:** LLn<br>**TAG:** L9-ONAC9:1/ OAzC9:1/ LSeC8:0/<br>LAzC9:0
649.46645	-1.46	$C_{38}H_{65}O_8^+$	$[M+H]^+$	**TAG:** LnAzC8:0/ LnSuC9:0
649.50311	-1.03	$C_{39}H_{69}O_7^+$	**DAG:** $[M+2O+H]^+$<br>**TAG:** $[M+H]^+$/ $[M+H]^+$/ $[M-H_2O+H]^+$/ [M-<br>$H_2O+H]^+$	**DAG:** LnO/ LL<br>**TAG:** S8-OOAC10:2/ O9-ONAC9:1/<br>OSeC8:0/ OAzC9:0
651.48391	1.32	$C_{38}H_{67}O_8^+$	$[M+H]^+$	**TAG:** LAzC8:0/ LSuC9:0/ PAzC10:2
659.48692	-1.83	$C_{40}H_{67}O_7^+$	$[M+H]^+$/ $[M-H_2O+H]^+$/ $[M-H_2O+H]^+$	**TAG:** L9-ONAC10:2/ LSeC9:1/ OAzC10:2
661.46796	0.85	$C_{39}H_{65}O_8^+$	**DAG:** $[M+3O+H]^+$<br>**TAG:** $[M+H]^+$	**DAG:** LnLn<br>**TAG:** LnAzC9:1/ LSuC10:2
663.48356	0.77	$C_{39}H_{67}O_8^+$	**DAG:** $[M+3O+H]^+$<br>**TAG:** $[M+H]^+$	**DAG:** LLn<br>**TAG:** LAzC9:1/ LnSeC8:0/ LnAzC9:0/<br>PoSeC10:2
665.49854	-0.24	$C_{39}H_{69}O_8^+$	**DAG:** $[M+3O+H]^+$<br>**TAG:** $[M+H]^+$	**DAG:** LnO/ LL<br>**TAG:** OAzC9:1/ LSeC8:0/ LAzC9:0/<br>PSeC10:2
673.50510	1.96	$C_{41}H_{69}O_7^+$	$[M-H_2O+H]^+$	**TAG:** LnSuC12:0/ LnSeC10:0/ OSeC10:2
677.49988	1.74	$C_{40}H_{69}O_8^+$	$[M+H]^+$	**TAG:** LSeC9:1
687.51758	-2.69	$C_{42}H_{71}O_7^+$	$[M-H_2O+H]^+$	**TAG:** LnAzC12:0/ LnMyrPi
689.49745	-1.81	$C_{41}H_{69}O_8^+$	$[M+H]^+$/ $[M-2H_2O+H]^+$	**TAG:** LSeC10:2/ OSeSe
703.51406	-0.41	$C_{42}H_{71}O_8^+$	$[M+H]^+$/$[M+O-H_2O+H]^+$/ $[M+O-H_2O+H]^+$	**TAG:** LnSeC11:1 /LnAzC12:0/ LnMyrPi
763.60643	-2.38	$C_{46}H_{83}O_8^+$	$[M+H]^+$	**TAG:** LPAz/ OOPi/ LSPi
787.60811	-0.18	$C_{48}H_{83}O_8^+$	$[M+H]^+$	**TAG:** LLAz/ LnOAz
789.62263	-1.61	$C_{48}H_{85}O_8^+$	$[M+H]^+$	**TAG:** LOAz/ LnSAz

Table S5. Interpretation of the mass spectrum of Prussian blue tempera (egg yolk) paint obtained with LA-APCI-FT-ICR-MS.

Abbreviations of fatty acids, dicarboxylic acids and other degradation products of triacylglycerols (**TAG**s), diacylglycerols (**DAG**s): **Ln** = linolenic acid ($C_{18}H_{30}O_2$); **L** = linoleic acid ($C_{18}H_{32}O_2$); **O** = oleic acid ($C_{18}H_{34}O_2$); **S** = stearic acid ($C_{18}H_{36}O2$); **P** = palmitic acid ($C_{16}H_{32}O_2$); **Po** = palmitoleic acid ($C_{16}H_{30}O_2$); **Eic** = eicosenoic acid ($C_{20}H_{38}O_2$); **C15:0** = pentadecanoic acid ($C_{15}H_{30}O_2$); **Myr** = myristic acid ($C_{14}H_{28}O_2$); **DDDA** = dodecanedioic acid ($C_{12}H_{22}O_4$); **Se** = sebacic acid ($C_{10}H_{18}O_4$); **Az** = azelaic acid ($C_9H_{16}O_4$); **9-ONA** = 9-oxononanoic acid ($C_9H_{16}O_3$); **C9:1** = 2-nonenoic acid ($C_9H_{16}O_2$); **C9:0** = nonanoic acid ($C_9H_{18}O_2$); **Su** = suberic acid ($C_8H_{14}O_4$); **8-OOA** = 8-oxooctanoic acid ($C_8H_{14}O_3$); **C8:1** = octenoic acid ($C_8H_{14}O_2$); **Pi** = pimelic acid ($C_7H_{12}O_4$). The abbreviation of monoacylglycerol is **MAG**.

Measured *m/z*	Δ*m/z* (ppm)	Ion formula		Possible compound (M)
257.24759	0.31	$C_{16}H_{33}O_2^+$	$[M+H]^+$	Palmitic acid
283.26352	1.27	$C_{18}H_{35}O_2^+$	$[M+H]^+$	Oleic acid
285.27938	2.00	$C_{18}H_{37}O_2^+$	$[M+H]^+$	Stearic acid
313.27414	1.34	$C_{19}H_{37}O_3^+$	$[M-H_2O+H]^+$	**MAG:** Glyceryl monopalmitate
339.29002	1.92	$C_{21}H_{39}O_3^+$	$[M-H_2O+H]^+$	**MAG:** Glyceryl monooleate
341.30551	1.44	$C_{21}H_{41}O_3^+$	$[M-H_2O+H]^+$	**MAG:** Glyceryl monostearate
367.33634	1.12	$C_{27}H_{43}^+$	$[M-H_2O+H]^+$	5-cholesten-3-one (cholesterol oxidation product)
369.35192	0.92	$C_{27}H_{45}^+$	$[M-H_2O+H]^+$	Cholesterol
383.33106	0.57	$C_{27}H_{43}O^+$	$[M+H]^+$	cholesta-4,6-diene-3-one (cholesterol oxidation product)
385.347	1.32	$C_{27}H_{45}O^+$	$[M+H]^+$/ $[M-H_2O+H]^+$	5-cholesten-3-one/ 5,6-epoxycholestan-3-ol or cholest-5-ene-3,7-diol (cholesterol oxidation product)
401.34198	1.42	$C_{27}H_{45}O_2^+$	$[M+H]^+$	3-hydroxycholest-5-en-7-one (cholesterol oxidation product)
467.37364	1.16	$C_{28}H_{51}O_5^+$	$[M+H]^+$/ $[M-H_2O+H]^+$	**DAG:** PoC9:1/ P9-ONA
479.37385	1.56	$C_{29}H_{51}O_5^+$	$[M+H]^+$/ $[M-H_2O+H]^+$/ $[M-2H_2O+H]^+$/ $[M-2H_2O+H]^+$	**DAG:** LC8:1/ O8-OOA/ SSu/ PSe
483.36889	1.80	$C_{28}H_{51}O_6^+$	$[M+H]^+$/ $[M-H_2O+H]^+$/ $[M-H_2O+H]^+$	**DAG:** Po9-ONA/ PAz/ SPi
493.38935	1.22	$C_{30}H_{53}O_5^+$	$[M+H]^+$/ $[M+H]^+$/ $[M-H_2O+H]^+$/ $[M-2H_2O+H]^+$	**DAG:** LC9:1/ LnC9:0/ O9-ONA/ SAz
495.40551	2.24	$C_{30}H_{55}O_5^+$	$[M+H]^+$/ $[M-H_2O+H]^+$/ $[M+H]^+$	**DAG:** OC9:1/ LC9:0/ S9-ONA
509.38468	1.98	$C_{30}H_{53}O_6^+$	$[M+H]^+$/ $[M-H_2O+H]^+$	**DAG:** L9-ONA/ OAz
523.47292	1.59	$C_{33}H_{63}O_4^+$	$[M-H_2O+H]^+$	**DAG:** PMyr
547.40031	1.81	$C_{33}H_{55}O_6^+$	$[M-H_2O+H]^+$	**DAG:** LnDDDA
547.4722	0.20	$C_{35}H_{63}O_4^+$	$[M-H_2O+H]^+$	**DAG**: PoPo/ LMyr
549.4887	1.75	$C_{35}H_{65}O_4^+$	$[M-H_2O+H]^+$	**DAG:** PPo/ OMyr
551.504	1.11	$C_{35}H_{67}O_4^+$	$[M-H_2O+H]^+$	**DAG:** PP/ SMyr
569.8324				Not assigned (NA)
573.48815	0.71	$C_{37}H_{65}O_4^+$	$[M-H_2O+H]^+$	**DAG:** LPo/ LnP
575.50362	0.40	$C_{37}H_{67}O_4^+$	$[M-H_2O+H]^+$	**DAG:** LP/ OPo
577.51872	-0.55	$C_{37}H_{69}O_4^+$	$[M-H_2O+H]^+$	**DAG:** OP/SPo
579.53383	-1.48	$C_{37}H_{71}O_4^+$	$[M-H_2O+H]^+$	**DAG:** SP
585.41599				NA
591.49912	1.39	$C_{37}H_{67}O_5^+$	$[M+H]^+$	**DAG**: LPo/ LnP
591.53511	0.71	$C_{38}H_{71}O_4^+$	$[M-H_2O+H]^+$	**DAG:** EicC15:0
593.51494	1.67	$C_{37}H_{69}O_5^+$	$[M+H]^+$	**DAG:** LP/ OPo
601.51944	0.66	$C_{39}H_{69}O_4^+$	$[M-H_2O+H]^+$	**DAG:** LO/ LnS
603.53442	-0.45	$C_{39}H_{71}O_4^+$	$[M-H_2O+H]^+$	**DAG:** LS/ OO
605.55109	1.24	$C_{39}H_{73}O_4^+$	$[M-H_2O+H]^+$	**DAG:** SO
611.98402				NA
615.49878	0.78	$C_{39}H_{67}O_5^+$	$[M+H]^+$	**DAG:** LLn
617.51512	1.89	$C_{39}H_{69}O_5^+$	$[M+H]^+$	**DAG:** LL/ OLn
619.53045	1.37	$C_{39}H_{71}O_5^+$	$[M+H]^+$	**DAG:** LO/ LnS
621.54558		$C_{39}H_{73}O_5^+$	$[M+H]^+$	**DAG:** LS/ OO
737.62836	-0.84	$C_{45}H_{85}O_7^+$	$[M+H]^+$	**TAG:** SP8-OOA
747.6123	-1.38	$C_{46}H_{83}O_7^+$	$[M+H]^+$	**TAG:** OPo9-ONA/ LnP9-ONA
749.62696	-2.69	$C_{46}H_{85}O_7^+$		**TAG:** SPo9-ONA/ OP9-ONA
757.63604	2.60	$C_{48}H_{85}O_6^+$	$[M+H]^+$	**TAG:** LOC9:1/ LnSC9:1
765.62687				NA
775.64539	0.98	$C_{48}H_{87}O_7^+$	$[M+H]^+$	**TAG:** LS9-ONA/ OO9-ONA

829.72897	1.21	$C_{53}H_{97}O_6^+$	$[M+H]^+$	**TAG:** LPoP/ OPoPo/ LnPP
831.74367	0.06	$C_{53}H_{99}O_6^+$	$[M+H]^+$	**TAG:** LPP/ OPoP/ SPoPo
833.75996	0.83	$C_{53}H_{101}O_6^+$	$[M+H]^+$	**TAG:** OPP/ SPoP
853.72782	-0.18	$C_{55}H_{97}O_6^+$	$[M+H]^+$	**TAG:** LLnP/ OPoLn/ LLPo
855.74362	0.00	$C_{55}H_{99}O_6^+$	$[M+H]^+$	**TAG:** LLP/ LOPo/ LnPO/ SLnPo
857.75849	-0.91	$C_{55}H_{101}O_6^+$	$[M+H]^+$	**TAG:** LSPo/ OOPo/ LnSP
859.77359	-1.55	$C_{55}H_{103}O_6^+$	$[M+H]^+$	**TAG:** LSP/ OOP/ SOPo
861.78469				NA
869.72294	0.07	$C_{55}H_{97}O_7^+$	$[M+O+H]^+$	**TAG:** LLPo /LLnP / LnOPo
871.7385	-0.03	$C_{55}H_{99}O_7^+$	$[M+O+H]^+$	**TAG:** LLP/ LOPo/ LnOP/ LnSPo
873.75373	-0.52	$C_{55}H_{10}1O_7^+$	$[M+O+H]^+$	**TAG:** LOP /LSPo / OOPo
875.76963	-0.23	$C_{55}H_{103}O_7^+$	$[M+O+H]^+$	**TAG:** LSP/ OOP/ SOPo
881.75987	0.68	$C_{57}H_{101}O_6^+$	$[M+H]^+$	**TAG:** LLO/ LLnS/ LnOO
883.77439	-0.60	$C_{57}H_{103}O_6^+$	$[M+H]^+$	**TAG:** LLS/ LOO
885.79013	-0.50	$C_{57}H_{105}O_6^+$	$[M+H]^+$	**TAG:** OOO/ LSO/ LnSS
887.73344	-0.01	$C_{55}H_{99}O_8^+$	$[M+2O+H]^+$	**TAG:** LLP/ LOPo/ LnOP/ LnSPo
887.80386	-2.66	$C_{57}H_{107}O_6^+$	$[M+H]^+$	**TAG:** OOS
888.75776				NA
889.76634				NA
897.75522	1.16	$C_{57}H_{101}O_7^+$	$[M+O+H]^+$	**TAG:** LLO/ LLnS/ LnOO
899.7689	-1.03	$C_{57}H_{103}O_7^+$	$[M+O+H]^+$	**TAG:** LOO/ LLS/ LnSO
900.77382				NA
901.77979				NA
902.7907				NA

Table S6. Interpretation of mass spectrum of matte dammar varnish obtained with LA-APCI-FT-ICR-MS.

Abbreviations of fatty acids of diacylglycerols (**DAGs**): **Ln** = linolenic acid ($C_{18}H_{30}O_2$); **L** = linoleic acid ($C_{18}H_{32}O_2$); **O** = oleic acid ($C_{18}H_{34}O_2$); **S** = stearic acid ($C_{18}H_{36}$O2); **P** = palmitic acid ($C_{16}H_{32}O_2$); **Po** = palmitoleic acid ($C_{16}H_{30}O_2$). Other abbreviation: NA – not assigned.

**Measured *m/z***	**Δ *m/z* (ppm)**	**Ion formula**		**Possible compound (M)**
141.794659				NA
147.12644				NA
177.16353	-1.41	$C_{13}H_{21}^+$		Unknown
191.17928	-0.76	$C_{14}H_{23}^+$		Unknown
203.17931	-0.60	$C_{15}H_{23}^+$		Unknown
217.19495	-0.58	$C_{16}H_{25}^+$		Unknown
231.21058	-0.65	$C_{17}H_{27}^+$		Unknown
249.19900				NA
299.27343	0.34	$C_{22}H_{35}^+$		Unknown
311.49779				NA
331.26322	0.18	$C_{22}H_{35}O_2^+$		Unknown
341.28403	0.40	$C_{24}H_{37}O^+$		Unknown
356.27962				NA
357.27846	-0.99	$C_{24}H_{37}O_2^+$		Unknown
391.33650	1.45	$C_{29}H_{43}^+$	$[M-H_2O-2H+H]^+$	Nor-amyrone ($C_{29}H_{46}O$) from dammar resin
393.31523	0.09	$C_{28}H_{41}O^+$		C28 fragment from dammar resin
393.35183	0.64	$C_{29}H_{45}^+$	$[M-H_2O+H]^+$	Nor-amyrone ($C_{29}H_{46}O$) from dammar resin
395.33115	0.78	$C_{28}H_{43}O^+$		C28 fragment of dammar resin
395.36764	1.02	$C_{29}H_{47}^+$	$[M-H_2O-CO-2H+H]^+$	Hydroxydammarenone ($C_{30}H_{50}O_2$) from dammar resin
405.31444	-1.86	$C_{29}H_{41}O^+$		C29 fragment of dammar resin
405.35159	0.01	$C_{30}H_{45}^+$	$[M-H_2O$-H2$+H]^+$	Dammaradienone ($C_{30}H_{48}O$) from dammar resin
407.33098	0.35	$C_{29}H_{43}O^+$	$[M-H_2O-2H+O+H]^+$	Nor-amyrone ($C_{29}H_{46}O$) from dammar resin
407.36726	0.08	$C_{30}H_{47}^+$	$[M-H_2O+H]^+$	Dammaradienone ($C_{30}H_{48}O$) from dammar resin
409.31086	1.83	$C_{28}H_{41}O_2^+$		C28 fragment of dammar resin
409.34658	0.21	$C_{29}H_{45}O^+$	$[M-2H+H]^+$	Nor-amyrone ($C_{29}H_{46}O$) from dammar resin
409.38285	-0.07	$C_{30}H_{49}^+$	$[M-H_2O+H]^+$	Dammaradienol ($C_{30}H_{50}O$) from dammar resin
411.36232	0.44	$C_{29}H_{47}O^+$	$[M+H]^+$	Nor-amyrone ($C_{29}H_{46}O$) from dammar resin
421.34670	0.49	$C_{30}H_{45}O^+$	$[M-H_2O+H]^+$	Oleanonic/ ursonic aldehyde ($C_{30}H_{46}O_2$) from dammar resin
423.32598	0.51	$C_{29}H_{43}O_2^+$	$[M-H_2O-2H+2O+H]^+$	Nor-amyrone ($C_{29}H_{46}O$) from dammar resin
423.36221	0.16	$C_{30}H_{47}O^+$	$[M-H_2O-2H+H]^+$	Hydroxydammarenone ($C_{30}H_{50}O_2$) from dammar resin
425.34166	0.58	$C_{29}H_{45}O_2^+$	$[M-2H+O+H]^+$	Nor-amyrone ($C_{29}H_{46}O$) from dammar resin
425.37769	-0.23	$C_{30}H_{49}O^+$	$[M+H]^+$	Dammaradienone ($C_{30}H_{48}O$) from dammar resin
427.35745	0.90	$C_{29}H_{47}O_2^+$	$[M+O+H]^+$	Nor-amyrone ($C_{29}H_{46}O$) from dammar resin
427.39399	1.29	$C_{30}H_{51}O^+$	$[M-H_2O+H]^+$	Dammarenediol ($C_{30}H_{52}O_2$) from dammar resin
437.34153	0.28	$C_{30}H_{45}O_2^+$	$[M-H_2O+H]^+$	Oleanonic/ ursonic acid ($C_{30}H_{46}O_3$) from dammar resin
439.32091	0.54	$C_{29}H_{43}O_3^+$	$[M-4H+2O+H]^+$	Nor-amyrone ($C_{29}H_{46}O$) from dammar resin
439.35716	0.23	$C_{30}H_{47}O_2^+$	$[M+H]^+$	Oleanonic/ ursonic aldehyde ($C_{30}H_{46}O_2$) from dammar resin
441.33682	1.14	$C_{29}H_{45}O_3^+$	$[M-2H+2O+H]^+$	Nor-amyrone ($C_{29}H_{46}O$) from dammar resin
441.37260	-0.26	$C_{30}H_{49}O_2^+$	$[M+O+H]^+$	Dammaradienone ($C_{30}H_{48}O$) from dammar resin
443.38825	-0.25	$C_{30}H_{51}O_2^+$	$[M+H]^+$	Hydroxydammarenone ($C_{30}H_{50}O_2$) from dammar resin
453.33677	1.00	$C_{30}H_{45}O_3^+$	$[M-2H+H]^+$	Oleanonic/ ursonic acid ($C_{30}H_{46}O_3$) from dammar resin
455.31577	0.40	$C_{29}H_{43}O_4^+$	$[M-4H+3O+H]^+$	Nor-amyrone ($C_{29}H_{46}O$) from dammar resin
455.35214	0.37	$C_{30}H_{47}O_3^+$	$[M+H]^+$	Oleanonic/ ursonic acid ($C_{30}H_{46}O_3$) from dammar resin
457.33102	-0.49	$C_{29}H_{45}O_4^+$	$[M-2H+3O+H]^+$	Nor-amyrone ($C_{29}H_{46}O$) from dammar resin
457.36788	0.57	$C_{30}H_{49}O_3^+$	$[M+H]^+$	Oleanolic/ ursolic acid ($C_{30}H_{48}O_3$) from dammar resin
469.33145	0.45	$C_{30}H_{45}O_4^+$	$[M-2H+O+H]^+$	Oleanonic/ ursonic acid ($C_{30}H_{46}O_3$) from dammar resin
471.34674	-0.32	$C_{30}H_{47}O_4^+$	$[M+O+H]^+$	Oleanonic/ ursonic acid ($C_{30}H_{46}O_3$) from dammar resin
509.43144				NA

577.51925	0.37	$C_{37}H_{69}O_4^+$	$[M-H_2O+H]^+$	DAG: OP/ SPo ($C_{37}H_{70}O_5$) - possibly from lead white oil paint under dammar varnish
601.52064	2.65	$C_{39}H_{69}O_4^+$	$[M-H_2O+H]^+$	DAG: LO/ LnS ($C_{39}H_{70}O_5$) - possibly from lead white oil paint under dammar varnish
603.53550	1.34	$C_{39}H_{71}O_4^+$	$[M-H_2O+H]^+$	DAG: LS/ OO ($C_{39}H_{72}O_5$) - possibly from lead white oil paint under dammar varnish
605.55079	0.73	$C_{39}H_{73}O_4^+$	$[M-H_2O+H]^+$	DAG: SO ($C_{39}H_{74}O_5$) - possibly from lead white oil paint under dammar varnish
611.55426	-1.27	$C_{45}H_{71}^+$	$[M+H]^+$	Combination of dammaradienone and cadinene ($C_{45}H_{70}$) from dammar resin
613.53489	0.97	$C_{44}H_{69}O^+$	$[M-H_2O-CO+O+H]^+$	Combination of oleanolic/ ursolic acid and cadinene ($C_{45}H_{70}O_2$) from dammar resin
613.57166	1.60	$C_{45}H_{73}^+$	$[M+H]^+$	Combination of dammaradienol and cadinene ($C_{45}H_{72}$) from dammar resin
615.55012	0.29	$C_{44}H_{71}O^+$	$[M-H_2O-CO+O+H]^+$	Combination of dammarenolic acid and cadinene ($C_{45}H_{72}O_2$) from dammar resin
625.53516	1.39	$C_{45}H_{69}O^+$	$[M+H]^+$	Combination of oleanonic/ ursonic aldehyde and cadinene ($C_{45}H_{68}O$) from dammar resin
627.55089	1.51	$C_{45}H_{71}O^+$	$[M+O+H]^+$	Combination of dammaradienone and cadinene ($C_{45}H_{70}$) from dammar resin
629.56657	1.55	$C_{45}H_{73}O^+$	$[M+H]^+$	Combination of hydroxydammarenone and cadinene ($C_{45}H_{72}O$) from dammar resin
641.53009	1.37	$C_{45}H_{69}O_2^+$	$[M+H]^+$	Combination of oleanonic/ ursonic acid and cadinene ($C_{45}H_{68}O_2$) from dammar resin
643.54608	1.90	$C_{45}H_{71}O_2^+$	$[M+H]^+$	Combination of oleanolic/ ursolic acid and cadinene ($C_{45}H_{70}O_2$) from dammar resin
645.56210	2.46	$C_{45}H_{73}O_2^+$	$[M+H]^+$	Combination of dammarenolic acid and cadinene ($C_{45}H_{72}O_2$) from dammar resin
647.57757	2.18	$C_{45}H_{75}O_2^+$	$[M+O+H]^+$	Combination of dammarenediol and cadinene ($C_{45}H_{74}O$) from dammar resin
659.53976	-0.02	$C_{45}H_{71}O_3^+$	$[M+O+H]^+$	Combination of oleanolic/ ursolic acid and cadinene ($C_{45}H_{70}O_2$) from dammar resin
661.55595	0.80	$C_{45}H_{73}O_3^+$	$[M+2O+H]^+$	Combination of hydroxydammarenone and cadinene ($C_{45}H_{72}O$) from dammar resin
675.70151	0.22	$C_{46}H_{91}O_2^+$	$[M+H]^+$	Monoester of triacontanol and palmitoleic acid ($C_{46}H_{90}O_2$) from beeswax
703.73286	0.28	$C_{48}H_{95}O_2^+$	$[M+H]^+$	Monoester of triacontanol and oleic acid ($C_{48}H_{94}O_2$) from beeswax
817.75989	1.72	$C_{60}H_{97}^+$	$[M+H]^+$	Combination of dammaradienol and 2 cadinene units ($C_{60}H_{96}$) from dammar resin
831.73772	-0.03	$C_{60}H_{95}O^+$	$[M+O+H]^+$	Combination of dammaradienone and 2 cadinene units ($C_{60}H_{94}$) from dammar resin
833.75388	0.59	$C_{60}H_{97}O^+$	$[M+H]^+$	Combination of hydroxydammarenone and 2 cadinene units ($C_{60}H_{96}O$) from dammar resin
835.74891				NA
847.73240	-0.31	$C_{60}H_{95}O_2^+$	$[M+H]^+$	Combination of oleanolic/ ursolic acid and 2 cadinene units ($C_{60}H_{94}O_2$) from dammar resin
847.84945	2.07	$C_{56}H_{111}O_4^+$	$[M+H]^+$	Diester of octacosane-1,28-diol with two myristic acids ($C_{56}H_{110}O_4$) from beeswax
849.74938	1.26	$C_{60}H_{97}O_2^+$	$[M+H]^+$	Combination of dammarenolic acid and 2 cadinene units ($C_{60}H_{96}O_2$) from dammar resin
863.72800	0.49	$C_{60}H_{95}O_3^+$	$[M+O+H]^+$	Combination of oleanolic/ ursolic acid and 2 cadinene units ($C_{60}H_{94}O_2$) from dammar resin
879.72051	-2.26	$C_{60}H_{95}O_4^+$	$[M+2O+H]^+$	Combination of oleanolic/ ursolic acid and 2 cadinene units ($C_{60}H_{94}O_2$) from dammar resin
903.90853	-1.95	$C_{60}H_{119}O_4^+$	$[M+H]^+$	Diester of triacontane-1,30-diol with palmitic acid and myristic acid ($C_{60}H_{118}O_4$) from beeswax

Measurement of extinction spectrum of lead white oil paint

The extinction (absorption and scattering) spectrum of lead white oil paint reference material was measured by Jasco V-570 (Japan) UV-VIS-IR spectrophotometer. The thin layer of the paint was deposited evenly to the silica plate by brush. The extinction coefficient was calculated by dividing the extinction value by the thickness of the material layer.

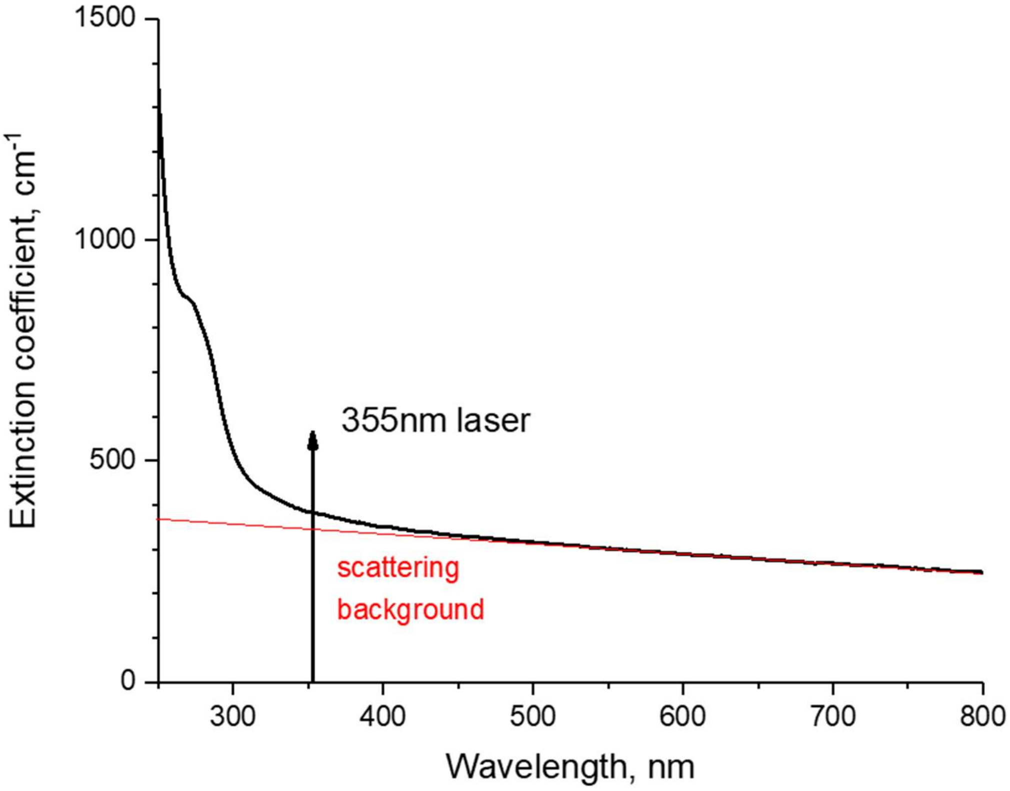

Figure S4. The spectrum of extinction coefficient of lead white oil paint. The contribution of scattering is significant. After the subtraction of the scattering background the absorption coefficient of lead white oil paint is estimated to be very low (~ 30 cm^{-1}) at 355 nm compared with copper resinate, which has an absorption coefficient of about 180 cm^{-1}.[8]

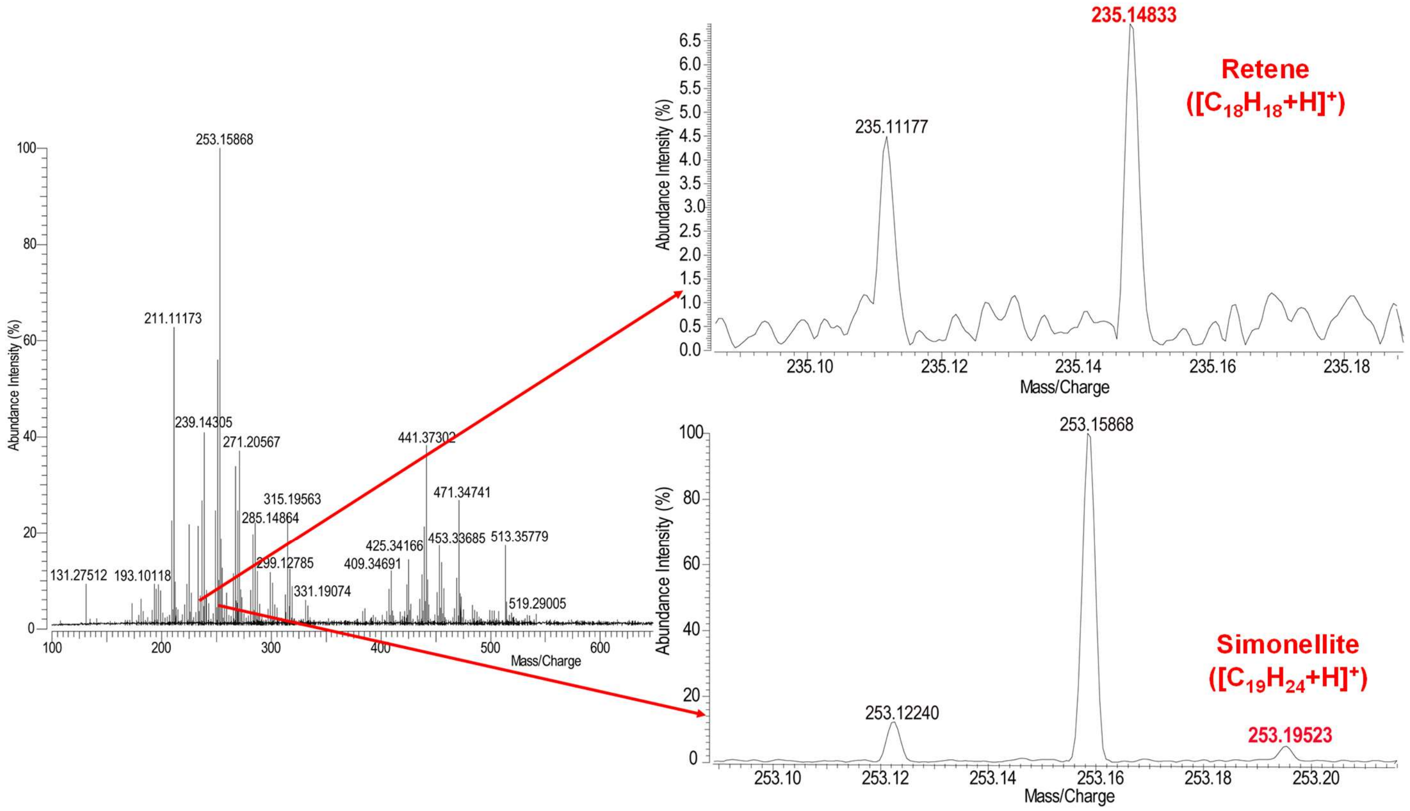

Figure S5. Mass spectrum of blackish brown material from an ointment jar from a 16th-century shipwreck obtained with LA-APCI-FT-ICR-MS. Zoomed are peaks of retene and simonellite (highlighted in red), which are marker compounds of pine tar.

Table S7. Interpretation of mass spectrum obtained from blackish brown material from an ointment jar from a 16th-century shipwreck with LA-APCI-FT-ICR-MS. Abbreviation: **NA** – not assigned.

**Measured *m/z***	**Δ *m/z* (ppm)**	**Ion formula**		**Possible compound (M)**
141.06986	-0.13	$C_{11}H_9^+$		Possibly pine tar
173.09588	-1.22	$C_{12}H_{13}O^+$		Possibly pine tar
173.13248	-0.02	$C_{13}H_{17}^+$		Possibly pine tar
179.08567	0.75	$C_{14}H_{11}^+$		Possibly pine tar
181.10116	-0.13	$C_{14}H_{13}^+$		Possibly pine tar
183.08056	0.64	$C_{13}H_{11}O^+$		Possibly pine tar
183.11679	-0.21	$C_{14}H_{15}^+$		Possibly pine tar
187.11172	-0.13	$C_{13}H_{15}O^+$		Possibly pine tar
191.17946	0.13	$C_{14}H_{23}^+$		Possibly pine tar
193.10118	0.00	$C_{15}H_{13}^+$		Possibly pine tar
195.08042	-0.13	$C_{14}H_{11}O^+$		Possibly pine tar
195.11682	-0.08	$C_{15}H_{15}^+$		Possibly pine tar
197.09611	0.12	$C_{14}H_{13}O^+$		Possibly pine tar
197.13248	0.01	$C_{15}H_{17}^+$		Possibly pine tar
199.07527	-0.48	$C_{13}H_{11}O_2^+$		Possibly pine tar
199.11176	0.10	$C_{14}H_{15}O^+$		Possibly pine tar
201.09094	-0.34	$C_{13}H_{13}O_2^+$		Possibly pine tar
205.10117	-0.07	$C_{16}H_{13}^+$		Possibly pine tar
207.08051	0.32	$C_{15}H_{11}O^+$		Possibly pine tar
209.09611	0.09	$C_{15}H_{13}O^+$		Possibly pine tar
209.13252	0.21	$C_{16}H_{17}^+$		Possibly pine tar
211.11173	-0.04	$C_{15}H_{15}O^+$		Possibly pine tar
213.09100	-0.04	$C_{14}H_{13}O_2^+$		Possibly pine tar
213.12731	-0.38	$C_{15}H_{17}O^+$		Possibly pine tar

215.10679	0.60	$C_{14}H_{15}O_2^+$		Possibly pine tar
219.11678	-0.22	$C_{17}H_{15}^+$		Possibly pine tar
221.13255	0.31	$C_{17}H_{17}^+$		Possibly pine tar
223.11170	-0.20	$C_{16}H_{15}O^+$		Possibly pine tar
223.14816	0.14	$C_{17}H_{19}^+$		Possibly pine tar
225.09111	0.45	$C_{15}H_{13}O_2^+$		Possibly pine tar
225.12741	0.10	$C_{16}H_{17}O^+$		Possibly pine tar
227.10676	0.42	$C_{15}H_{15}O_2^+$		Possibly pine tar
227.14313	0.41	$C_{16}H_{19}O^+$		Possibly pine tar
231.11678	-0.20	$C_{18}H_{15}^+$		Possibly pine tar
233.13249	0.03	$C_{18}H_{17}^+$		Possibly pine tar
235.11177	0.11	$C_{17}H_{15}O^+$		Possibly pine tar
235.14833	0.83	$C_{18}H_{19}^+$	$[M+H]^+$	Retene ($C_{18}H_{18}$) from pine tar
237.12747	0.34	$C_{17}H_{17}O^+$		Possibly pine tar
237.16381	0.13	$C_{18}H_{21}^+$		Possibly pine tar
239.10671	0.22	$C_{16}H_{15}O_2^+$		Possibly pine tar
239.14305	0.05	$C_{17}H_{19}O^+$		Possibly pine tar
241.12235	0.15	$C_{16}H_{17}O_2^+$		Possibly pine tar
241.15851	-0.73	$C_{17}H_{21}O^+$		Possibly pine tar
243.10184	1.10	$C_{15}H_{15}O_3^+$		Possibly pine tar
243.13795	-0.03	$C_{16}H_{19}O_2^+$		Possibly pine tar
247.11172	-0.06	$C_{18}H_{15}O^+$		Possibly pine tar
249.12741	0.06	$C_{18}H_{17}O^+$		Possibly pine tar
251.10658	-0.31	$C_{17}H_{15}O_2^+$		Possibly pine tar
251.14304	0.00	$C_{18}H_{19}O^+$		Possibly pine tar
251.17964	0.84	$C_{19}H_{23}^+$	$[M-H_2O-CO-2H+H]^+$	Didehydroabietic acid ($C_{20}H_{26}O_2$)
253.12240	0.34	$C_{17}H_{17}O_2^+$		Possibly pine tar
253.15868	-0.05	$C_{18}H_{21}O^+$		Possibly pine tar
253.19523	0.58	$C_{19}H_{25}^+$	$[M+H]^+$	Simonellite ($C_{19}H_{24}$) from pine tar
255.10155	-0.08	$C_{16}H_{15}O_3^+$		Possibly pine tar
255.13803	0.27	$C_{17}H_{19}O_2^+$		Possibly pine tar
255.17451	0.68	$C_{18}H_{23}O^+$		Possibly pine tar
255.21084	0.41	$C_{19}H_{27}^+$	$[M-H_2O-CO+H]^+$	Dehydroabietic acid ($C_{20}H_{28}O_2$)
257.15380	0.73	$C_{17}H_{21}O_2^+$		Possibly pine tar
257.18996	-0.13	$C_{18}H_{25}O^+$		Possibly pine tar
257.22650	0.45	$C_{19}H_{29}^+$	$[M+H]^+$	Nor-abietatriene ($C_{19}H_{28}$) from pine tar
259.24222	0.71	$C_{19}H_{31}^+$		Possibly pine tar
261.25797	1.11	$C_{19}H_{33}^+$		Possibly pine tar
263.14296	-0.30	$C_{19}H_{19}O^+$		Possibly pine tar
265.12242	0.43	$C_{18}H_{17}O_2^+$		Possibly pine tar
265.15902	1.23	$C_{19}H_{21}O^+$		Possibly pine tar
267.13799	0.10	$C_{18}H_{19}O_2^+$		Possibly pine tar
267.17438	0.14	$C_{19}H_{23}O^+$	$[M-H_2O-CO-2H+H]^+$	7-oxodehydroabietic acid ($C_{20}H_{26}O_3$)
269.11731	0.32	$C_{17}H_{17}O_3^+$		Possibly pine tar
269.15372	0.42	$C_{18}H_{21}O_2^+$		Possibly pine tar
269.19013	0.50	$C_{19}H_{25}O^+$	$[M-H_2O-CO+H]^+$	7-oxodehydroabietic acid ($C_{20}H_{26}O_3$)
271.13302	0.53	$C_{17}H_{19}O_3^+$		Possibly pine tar
271.16934	0.28	$C_{18}H_{23}O_2^+$		Possibly pine tar
271.20567	0.10	$C_{19}H_{27}O^+$	$[M-H_2O-CO+H]^+$	15-hydroxydehydroabietic acid ($C_{20}H_{28}O_3$)
273.14883	1.13	$C_{17}H_{21}O_3^+$		Possibly pine tar
273.22131	0.07	$C_{19}H_{29}O^+$	$[M-H_2O-CO+O+H]^+$	Abetic acid ($C_{20}H_{30}O_2$)
275.23707	0.47	$C_{19}H_{31}O^+$		Possibly pine tar
279.13792	-0.14	$C_{19}H_{19}O_2^+$		Possibly pine tar
281.11735	0.47	$C_{18}H_{17}O_3^+$		Possibly pine tar
281.15359	-0.09	$C_{19}H_{21}O_2^+$		Possibly pine tar
283.13303	0.58	$C_{18}H_{19}O_3^+$		Possibly pine tar
283.16938	0.42	$C_{19}H_{23}O_2^+$	$[M-H_2O-CO-2H+H]^+$	15-hydroxy-7-oxodehydroabietic acid ($C_{20}H_{26}O_4$)
285.14864	0.41	$C_{18}H_{21}O_3^+$		Possibly pine tar
285.18508	0.61	$C_{19}H_{25}O_2^+$	$[M-H_2O-CO+H]^+$	15-hydroxy-7-oxodehydroabietic acid ($C_{20}H_{26}O_4$)
285.22132	0.10	$C_{20}H_{29}O^+$	$[M-H_2O+H]^+$	Abietic acid ($C_{20}H_{30}O_2$)
287.16401	-0.54	$C_{18}H_{23}O_3^+$		Possibly pine tar
287.20068	0.40	$C_{19}H_{27}O_2^+$	$[M-H_2O-CO+H]^+$	7,15-dihydroxydehydroabietic acid ($C_{20}H_{28}O_4$)
287.23715	0.73	$C_{20}H_{31}O^+$		Possibly pine tar
289.21642	0.73	$C_{19}H_{29}O_2^+$	$[M-H_2O-CO+2O+H]^+$	Abietic acid ($C_{20}H_{30}O_2$)
297.11230	0.54	$C_{18}H_{17}O_4^+$		Possibly pine tar

297.14877	0.85	$C_{19}H_{21}O_3^+$		Possibly pine tar
297.18501	0.32	$C_{20}H_{25}O_2^+$	$[M-H_2O+H]^+$	7-oxodehydroabietic acid ($C_{20}H_{26}O_3$)
299.12785	0.20	$C_{18}H_{19}O_4^+$		Possibly pine tar
299.16413	-0.15	$C_{19}H_{23}O_3^+$	$[M-H_2O-CO-2H+O+H]^+$	15-hydroxy-7-oxodehydroabietic acid ($C_{20}H_{26}O_4$)
299.20077	0.69	$C_{20}H_{27}O_2^+$	$[M+H]^+$	Didehydroabietic acid ($C_{20}H_{26}O_2$)
301.14383	1.31	$C_{18}H_{21}O_4^+$		Possibly pine tar
301.18041	1.95	$C_{19}H_{25}O_3^+$	$[M-H_2O-CO+O+H]^+$	15-hydroxy-7-oxodehydroabietic acid ($C_{20}H_{26}O_4$)
301.21636	0.49	$C_{20}H_{29}O_2^+$	$[M+H]^+$	Dehydroabietic acid ($C_{20}H_{28}O_2$) from pine tar
303.19583	1.18	$C_{19}H_{27}O_3^+$	$[M-H_2O-CO+O+H]^+$	7,15-dihydroxydehydroabietic acid ($C_{20}H_{28}O_4$)
303.23213	0.89	$C_{20}H_{31}O_2^+$	$[M+H]^+$	Abietic acid ($C_{20}H_{30}O_2$) from pine tar
305.21119	0.22	$C_{19}H_{29}O_3^+$	$[M-H_2O-CO+3O+H]^+$	Abietic acid ($C_{20}H_{30}O_2$)
305.24769	0.58	$C_{20}H_{33}O_2^+$		Possibly pine tar
313.02676				NA
313.07247				NA
313.11797				NA
313.16379	-2.46	$C_{16}H_{25}O_6^+$		Possibly pine tar
313.17979	-0.08	$C_{20}H_{25}O_3^+$	$[M-H_2O+H]^+$	15-hydroxy-7-oxodehydroabietic acid ($C_{20}H_{26}O_4$)
315.19563	0.49	$C_{20}H_{27}O_3^+$	$[M+H]^+$	7-oxodehydroabietic acid ($C_{20}H_{26}O_3$)
317.21135	0.71	$C_{20}H_{29}O_3^+$	$[M+H]^+$	15-hydroxydehydroabietic acid ($C_{20}H_{28}O_3$)
319.22695	0.56	$C_{20}H_{31}O_3^+$	$[M+O+H]^+$	Abietic acid ($C_{20}H_{30}O_2$)
331.19074	1.06	$C_{20}H_{27}O_4^+$	$[M+H]^+$	15-hydroxy-7-oxodehydroabietic acid ($C_{20}H_{26}O_4$)
333.20614	0.30	$C_{20}H_{29}O_4^+$	$[M+H]^+$	7,15-dihydroxydehydroabietic acid ($C_{20}H_{28}O_4$)
383.33134	1.30	$C_{27}H_{43}O^+$		Unknown
385.34738	2.31	$C_{27}H_{45}O^+$		Unknown
393.35184	0.67	$C_{29}H_{45}^+$		Triterpene or triterpenoid
401.34206	1.62	$C_{27}H_{45}O_2^+$		Unknown
405.31677				NA
407.33132	1.18	$C_{29}H_{43}O^+$		Triterpenoid
407.36710	-0.33	$C_{30}H_{47}^+$		Triterpene or triterpenoid
409.34691	1.04	$C_{29}H_{45}O^+$		Triterpenoid
409.38342	1.31	$C_{30}H_{49}^+$		Triterpene or triterpenoid
417.48813				NA
421.31019	0.19	$C_{29}H_{41}O_2^+$		Triterpenoid
421.34621	-0.67	$C_{30}H_{45}O^+$		Triterpenoid
423.32629	1.26	$C_{29}H_{43}O_2^+$		Triterpenoid
423.36260	1.09	$C_{30}H_{47}O^+$		Triterpenoid
425.34166	0.58	$C_{29}H_{45}O_2^+$		Triterpenoid
425.37829	1.17	$C_{30}H_{49}O^+$		Triterpenoid
427.35751	1.04	$C_{29}H_{47}O_2^+$		Triterpenoid
435.28913	-0.55	$C_{29}H_{39}O_3^+$		Triterpenoid
435.32624	1.10	$C_{30}H_{43}O_2^+$		Triterpenoid
437.30557	1.26	$C_{29}H_{41}O_3^+$		Triterpenoid
437.34166	0.58	$C_{30}H_{45}O_2^+$		Triterpenoid
439.32158	2.07	$C_{29}H_{43}O_3^+$		Triterpenoid
439.35775	1.57	$C_{30}H_{47}O_2^+$		Triterpenoid
441.33674	0.94	$C_{29}H_{45}O_3^+$		Triterpenoid
441.37302	0.70	$C_{30}H_{49}O_2^+$		Triterpenoid
443.35240	0.97	$C_{29}H_{47}O_3^+$		Triterpenoid
451.32110	0.96	$C_{30}H_{43}O_3^+$		Triterpenoid
453.29970	-0.53	$C_{29}H_{41}O_4^+$		Triterpenoid
453.33685	1.16	$C_{30}H_{45}O_3^+$		Triterpenoid
455.31718				NA
455.35231	0.75	$C_{30}H_{47}O_3^+$		Triterpenoid
457.33151	0.59	$C_{29}H_{45}O_4^+$		Triterpenoid
457.36790	0.60	$C_{30}H_{49}O_3^+$		Triterpenoid
467.31531	-0.61	$C_{30}H_{43}O_4^+$		Triterpenoid
469.33150	0.56	$C_{30}H_{45}O_4^+$		Triterpenoid
471.34741	1.11	$C_{30}H_{47}O_4^+$		Triterpenoid
473.36275	0.44	$C_{30}H_{49}O_4^+$		Triterpenoid
475.37983				NA
483.38391	1.32	$C_{32}H_{51}O_3^+$		Unknown
485.28465	1.56	$C_{36}H_{37}O^+$		Unknown
485.32683	1.40	$C_{30}H_{45}O_5^+$		Triterpenoid
487.29872	-1.68	$C_{36}H_{39}O^+$		Unknown
487.34260	1.65	$C_{30}H_{47}O_5^+$		Triterpenoid

499.26351	0.70	$C_{36}H_{35}O_2^+$		Unknown
501.27903	0.44	$C_{36}H_{37}O_2^+$		Unknown
503.29378	-1.35	$C_{36}H_{39}O_2^+$		Unknown
507.28853	-1.67	$C_{35}H_{39}O_3^+$		Unknown
513.35779	0.66	$C_{32}H_{49}O_5^+$		Unknown
519.29005	1.31	$C_{36}H_{39}O_3^+$		Unknown
541.40389	-0.21	$C_{38}H_{53}O_2^+$		Unknown
663.45529	-1.13	$C_{49}H_{59}O^+$		Unknown

References

(1) Teearu, A.; Vahur, S.; Rodima, T.; Herodes, K.; Bonrath, W.; Netscher, T.; Tshepelevitsh, S.; Trummal, A.; Lõkov, M.; Leito, I. Method Development for the Analysis of Resinous Materials with MALDI-FT-ICR-MS: Novel Internal Standards and a New Matrix Material for Negative Ion Mode. *J. Mass Spectrom.* **2017**, *52* (9), 603–617. https://doi.org/10.1002/jms.3943.

(2) Tammekivi, E.; Vahur, S.; Vilbaste, M.; Leito, I. Quantitative GC–MS Analysis of Artificially Aged Paints with Variable Pigment and Linseed Oil Ratios. *Molecules* **2021**, *26* (8), 2218. https://doi.org/10.3390/molecules26082218.

(3) *Organic Mass Spectrometry in Art and Archaeology*; Colombini, M. P., Modugno, F., Eds.; Wiley: Chichester, West Sussex, 2009.

(4) Tirat, S.; Degano, I.; Echard, J.-P.; Lattuati-Derieux, A.; Lluveras-Tenorio, A.; Marie, A.; Serfaty, S.; Le Huerou, J.-Y. Historical Linseed Oil/Colophony Varnishes Formulations: Study of Their Molecular Composition with Micro-Chemical Chromatographic Techniques. *Microchem. J.* **2016**, *126*, 200–213. https://doi.org/10.1016/j.microc.2015.11.045.

(5) Wei, S.; Schreiner, M.; Rosenberg, E.; Guo, H.; Ma, Q. The Identification of Binding Media in the Tang Dynasty Chinese Wall Paintings by Using Py-GC/MS and GC/MS Techniques. *Int. J. Conserv. Sci.* **2011**, *2* (2), 77–88.

(6) van den Berg, J. D. J.; van den Berg, K. J.; Boon, J. J. Identification of Non-Cross-Linked Compounds in Methanolic Extracts of Cured and Aged Linseed Oil-Based Paint Films Using Gas Chromatography–Mass Spectrometry. *J. Chromatogr. A* **2002**, *950* (1), 195–211. https://doi.org/10.1016/S0021-9673(02)00049-3.

(7) Mills, J. S.; White, R. *The Organic Chemistry of Museum Objects*, 2. ed., reprinted.; Butterworth-Heinemann series in conservation and museology; Butterworth-Heinemann: Oxford, 2003.

(8) Vahur, S.; Teearu, A.; Lohmus, R.; Leissoo, M.; Treshchalov, A.; Lungevics, J.; Arju, G.; Hiiop, H. Characterisation of Laser-Ablated Craters of Different Painting Materials and Evaluation with Modified LA-APCI-MS System. *Talanta* **2025**, *291*, 127856. https://doi.org/10.1016/j.talanta.2025.127856.
